# Comprehensive analysis of Hibisci mutabilis Folium extract’s mechanisms in alleviating UV-induced skin photoaging through enhanced network pharmacology and experimental validation

**DOI:** 10.3389/fphar.2024.1431391

**Published:** 2024-10-14

**Authors:** Wenyuan Chen, Qin Deng, Bili Deng, Yueping Li, Gengqi Fan, Fangfang Yang, Wei Han, Jian Xu, Xiaolan Chen

**Affiliations:** ^1^ Department of Pharmacy, Guizhou University of Traditional Chinese Medicine, Guiyang, Guizhou, China; ^2^ School of Pharmacy, Bijie Medical College, Bijie, Guizhou, China; ^3^ Guizhou provincial Institute for Food Inspection, Guiyang, Guizhou, China

**Keywords:** Hibisci mutabilis Folium, traditional Chinese medicine, skin photoaging, ultraviolet irradiation, network pharmacology, inflammation, mechanism

## Abstract

**Background:**

Skin photoaging induced by ultraviolet A (UVA) and ultraviolet B (UVB) radiation manifests as skin roughness, desquamation, pigmentation, and wrinkle formation. Current treatments, such as sunscreen, hormones, and antioxidants, have limitations and side effects. Traditional Chinese Medicine Hibisci Mutabilis Folium (HMF), or Mu-Fu-Rong-Ye in Chinese name, refers to the dried leaves of the plant *Hibiscus mutabilis* L., which belongs to the Malvaceae family. It has been used traditionally to treat acute mastitis, parotitis, neurodermatitis, burns. The reported activities of HMF include anti-inflammatory and anti-oxidant effects. However, the therapeutic potential of HMF in preventing and treating UV-induced skin photoaging remains unexplored.

**Objective:**

This study aimed to investigate the protective effects of HMF extract (EHMF) against UV-induced skin photoaging and the underlying mechanisms of action, by using network pharmacology and experimental verification.

**Methods:**

Network pharmacology was employed to identify the effective chemical components of EHMF. Potential targets were identified via PPI network analysis. Representative compounds were characterized using UPLC-MS/MS. *In vitro* validation involved assessing HaCaT cell viability, observing live/dead cell staining through fluorescence microscopy, and measuring inflammatory factors using ELISA. For *in vivo* validation, a UV-induced skin photoaging mice model was treated transdermally with EHMF or Methotrexate daily for 7 days. Dermatitis severity, skin morphology, and collagen fiber pathology were evaluated. Inflammatory cytokine and protein expression in dorsal skin lesions was confirmed using Elisa Kits, Western blot and immunohistochemistry.

**Results:**

A total of 22 active ingredients of EHMF were identified. GO enrichment and KEGG pathway analyses revealed a focus on inflammatory signaling pathways. *In vitro* experiments showed that EHMF significantly reduced UV-induced inflammatory factors in HaCaT cells and improved cell survival rates. *In vivo*, EHMF alleviated back skin lesions in UV-exposed mice, reducing epidermal and dermal thickening and pathological inflammatory cell infiltration. It also decreased abnormal MMP-9 expression and collagen fiber proliferation, along with levels of inflammatory factors like TNF-α, IL-6, IL-17, and EGFR. Western blot and immunohistochemistry results indicated that the over-activation of the AKT-STAT3 signaling pathway was inhibited.

**Conclusion:**

EHMF effectively reduced UV-induced skin damage, inflammation, and wrinkles, providing strong support for its clinical application as a dermatological agent.

## Graphical Abstract

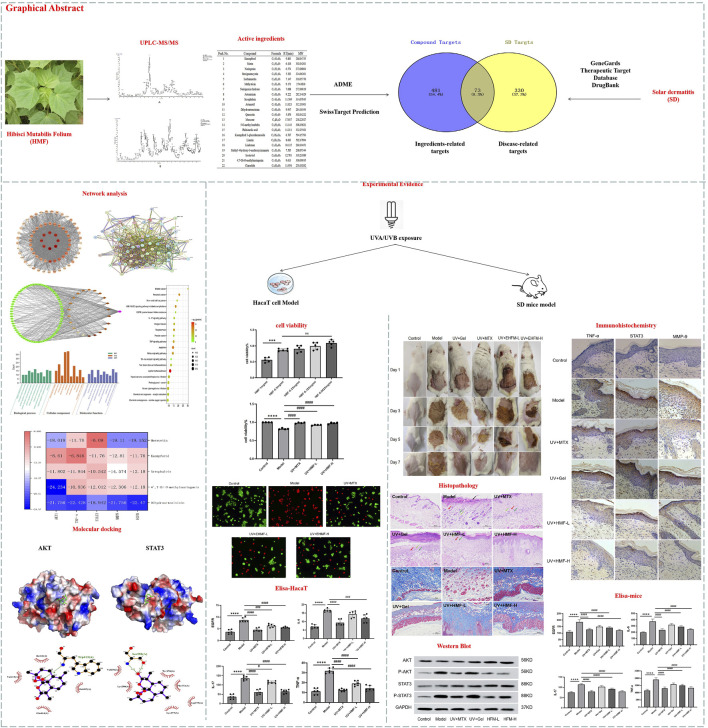

## 1 Introduction

Skin photoaging refers to kinase cascades triggered (Xiao J. et al., 2022) by epidermal growth factor and cytokine receptor activation under ultraviolet (UV) radiation, leading to reduced skin elasticity, roughness, and wrinkle formation (Camponogara and Oliveira, 2022; Garnacho Saucedo et al., 2020; Zhu et al., 2022). Kinase cascades triggered denotes a series of activation reactions of kinases in the process of intracellular signal transduction. These kinases sequentially phosphorylate downstream proteins, creating a cascading effect in signal transduction that amplifies the signal and regulates various biological responses in the cell, such as growth, differentiation, and response to external stimuli. UV radiation is the primary external cause of skin photoaging, followed by environmental pollutants (de Gruijl, 2017). UVA (315–400 nm) and UVB (280–315 nm) are the main contributors to UV-induced damage, which involves inflammation, DNA damage, oxidative stress, immunosuppression, apoptosis, and aging (Andrade et al., 2022). Clinically, UV-induced skin photoaging is characterized by depigmentation, mottled pigmentation, epidermal thickening, and increased wrinkles, often linked to acne, seborrheic keratosis, pigmented spots, and skin tumors (Kapadia et al., 2003; Swiader et al., 2021). UV-induced skin photoaging imposes psychological and social burdens on patients, and associated conditions like skin tumors pose serious public health concerns (Kolitz et al., 2022). Sunscreens and skincare products offer UV protection but have limited efficacy in reversing skin photoaging (Guan et al., 2021; Liang et al., 2022; Shin et al., 2019). Retinoic acid, 5-fluorouracil, and antioxidants can reverse photodamaged skin to some extent but often have side effects and usage restrictions (Milosheska and Roškar, 2022; Searle et al., 2021).

Hibisci Mutabilis Folium (HMF), with the Chinese name of Mu-Fu-Rong-Ye, also known as Ju-Shuang-Ye, refers to the dried leaves of the *Hibiscus mutabilis* L. plant, which belongs to the Malvaceae family. As a traditional Chinese medicinal material, HMF was first recorded in the “Ben Cao Tu Jing” and has a long history of medicinal use. HMF is used in TCM to treat herpes zoster, swelling, and bruising (Searle et al., 2021). It is widely distributed in China and also cultivated in Japan and Southeast Asia. “Ben Cao Tu Jing” (Illustration of Materia Medica) describes HMF as “congealing and poisoning.” In minority areas of China, it is also used to treat pneumonia, otitis media, appendicitis, rhinitis, and lymphadenitis. According to the 2020 edition of the Chinese Pharmacopoeia, HMF is harvested in summer and autumn and dried using various methods (Wu et al., 2022). It is often decocted or powdered for direct application. Modern research identifies flavonoids, organic acids, coumarins, and triterpene acids as its main components (Abdelhafez et al., 2020; Kumar et al., 2012; Liu et al., 2015; Xu et al., 2019), with biological activities like antioxidant (He et al., 2023), anti-infection, antibacterial, and antiviral properties (Xiao M. et al., 2023).

TCM offers advantages like fewer side effects and stable performance. Research shows that many Chinese medicinal herbs can improve collagen repair by scavenging free radicals (Vargas-Ruiz et al., 2023), resisting oxidative stress (Chen S. et al., 2023), and inhibiting inflammation (Chen Y. et al., 2023) and apoptosis (Petruk et al., 2018). Ginseng, a common cosmetic ingredient due to ginsenosides, has antioxidant, anti-aging, and anti-inflammatory properties. Red ginseng improves facial wrinkles and enhances type I procollagen synthesis (Flament et al., 2019b). Resveratrol enhances superoxide dismutase (SOD) and glutathione peroxidase (GSH-Px) activity while reducing malondialdehyde (MDA) after UVB exposure (Bosch et al., 2015). Catechins in tea inhibit UV-activated matrix metalloproteinases and reduce cyclobutane pyrimidine dimers. A study found that 300 mg of green tea twice daily, combined with 10% green tea cream, increased skin elasticity (Flament et al., 2019a). Puerarin, extracted from pueraria roots, effectively scavenges free radicals (Flament et al., 2015). The complex chemical composition of TCM makes traditional pharmacological study challenging. Network pharmacology (Cheung et al., 2024), based on disease-related targets and protein interaction networks, reveals core targets and mechanisms, marking a shift from “composition” to “target-network-drug effect”.

Currently, The reported activities of EHMF include anti-inflammatory and anti-oxidant effects. However, the therapeutic potential of HMF in preventing and treating UV-induced skin photoaging remains unexplored. In order to reveal the potential molecular mechanisms and pathways of EHMF in the treatment of UV-induced skin photoaging, we utilized UPLC-MS/MS to identify EHMF components and network pharmacology to screen potential UV-induced skin photoaging targets. Molecular docking technology explored the mechanisms, and *in vitro* and *in vivo* experiments clarified EHMF’s pharmacological effects, providing theoretical evidence for its clinical application in UV-induced skin photoaging.

## 2 Materials and methods

### 2.1 Extraction of EHMF

The HMF samples were sourced from Guangxi Yulin Chinese Herbs Co., Ltd. and authenticated by Professor Ding Ning from the Guizhou University of Traditional Chinese Medicine.

The preparation method was consulted with a previous study (Wang et al., 2014). In brief, Weighed 200 g of HMF crude powder. Extracted with 50% ethanol using a solid-liquid ratio of 1:14 via reflux for 30 min. Repeated extraction two more times, combining all extracts. Filtered and concentrated the extracts to a 0.2 g/mL concentration. Purified the concentrated solution using polyamide and macroporous resins. Stored the final extract at 4°C for stability.

### 2.2 UPLC-MS/MS analysis

Samples of 100 mg were subjected to sonication at a low temperature (40 kHz) for 10 min following the addition of 1 mL of 70% methanol. The mixtures were then centrifuged at 12,000 rpm for 10 min at 4°C, and the resulting supernatants were diluted 50-fold and supplemented with 10 μL of an internal standard solution (100 μg/mL). Subsequently, the samples were filtered through a 0.22 μm PTFE filter. Analytical determination was conducted using a Thermo Vanquish Q Exactive HF LC-MS system equipped with a Zorbax Eclipse C18 column (1.8 μm × 2.1 mm × 100 mm). The instrument settings were as follows: heater temperature at 325°C, sheath gas flow at 45 arbitrary units, auxiliary gas flow at 15 arbitrary units, and purge gas flow at one arbitrary unit. The electrospray voltage was set to 3.5 kV, and the capillary temperature was maintained at 330°C, with an S-Lens RF Level of 55%. Chromatographic conditions included a column temperature of 30°C, a mobile phase of 0.1% formic acid in water (A) and pure acetonitrile (B), and a sample injection volume of 2 μL at an autosampler temperature of 4 °C. The flow rate was set to 0.3 mL/min with a gradient program of 5% B from 0–2 min, increasing to 30% B over 2–70 min, then to 78% B from 7–14 min, reaching 95% B by 14–20 min, and returning to 5% B from 20–25 min. All data were processed using Compound Discoverer 3.3, with substance identification performed using the Thermo mzCloud online database and the Thermo mzVault local database, based on secondary mass spectrometry data.

### 2.3 Network pharmacological analysis

#### 2.3.1 Target prediction

The targets of each active component identified in EHMF via UPLC-MS/MS analysis were retrieved from the TCMSP database (https://old.tcmsp-e.com/tcmsp.php). For those active compounds lacking target information in the TCMSP database, their two-dimensional structural formulas were obtained from the PubChem database (https://pubchem.ncbi.nlm.nih.gov). Potential targets were then predicted using the Swiss Target Prediction server, restricting predictions to the human species. The names of these targets were standardized by referencing the UniProt database (https://www.uniprot.org).

#### 2.3.2 Screening of potential targets for UV-induced skin photoaging

To focus our investigation on UV-induced skin photoaging, we utilized “photoaging” as a search keyword to extract relevant target information from the GeneCards database. This selection was refined to include only targets with correlation coefficients above the median value and those possessing significant gene-disease association scores. This methodology ensured a rigorous selection process, tailored to enhance our understanding of the molecular interactions relevant to UV-induced skin photoaging.

#### 2.3.3 PPI network analysis

Utilizing the Draw Venn Diagram tool, we integrated the disease-specific targets with those associated with the medication to delineate potential targets of EHMF in combating UV-induced skin photoaging. Subsequently, a PPI network was constructed via the STRING database (https://string-db.org/, version 11.0), confined to the species *Homo sapiens* and a stringent confidence threshold of 1.0. Nodes lacking connectivity were systematically excluded to ensure network coherence. The network’s topology was thoroughly analyzed using the CytoNCA plug-in within the Cytoscape 3.8.2 software. Based on this analysis, the top 10 targets exhibiting the highest node degree values were identified as core targets, earmarked for further investigation through molecular docking studies. This approach facilitated a targeted and systematic exploration of the molecular mechanisms through which EHMF may exert therapeutic effects against UV-induced skin photoaging.

#### 2.3.4 Enrichment analysis

Gene Ontology (GO) and Kyoto Encyclopedia of Genes and Genomes (KEGG) pathway enrichment analyses were performed using R language programming to elucidate the functional roles and signaling pathways involved in pharmacological therapy. Initially, primary annotation data were rigorously filtered to ensure data integrity. The GO analysis was stratified into three categories: biological processes (BP), cellular components (CC), and molecular functions (MF), each providing insights into the different functional aspects of the target proteins. Concurrently, KEGG analysis was employed to identify and characterize the principal signaling pathways associated with the pharmacological treatment of diseases, enabling a deeper understanding of the molecular mechanisms underpinning therapeutic effects. This methodological approach ensures a comprehensive analysis of the functional and pathway-related implications of target proteins in disease treatment.

#### 2.3.5 Docking analysis

The compounds exhibiting the top five degree values in the EHMF active ingredient-target network were chosen as ligands for further study. Their three-dimensional structures were obtained from the PubChem database. Similarly, the targets with the highest degree values were identified in both the drug-disease common target PPI network and the principal pathway-target network. Protein crystal structures were sourced from the AlphaFold database to ensure accuracy in molecular modeling.

Prior to docking, necessary modifications to the receptor proteins and chemical ligands were made using the AutoDockTools software to optimize interaction predictions. The molecular docking was subsequently performed using the AutoDock program, facilitating the prediction of binding affinities and interaction configurations between the ligands and their respective targets. The resulting docking conformations were then visualized in PyMOL2.5, providing a detailed and interpretable representation of the molecular interactions.

### 2.4 Cell experimentals verification

HaCaT cells were obtained from Wuhan Zisan Biotechnology Co., Ltd. and cultured in DMEM supplemented with 10% fetal bovine serum (FBS; Gibco, USA), penicillin (100 U/ml), and streptomycin (100 μg/mL) at 37°C in a humidified 5% CO_2_ atmosphere. The experimental design included five groups: Control group, Model group exposed to UV radiation, UV + MTX group treated with 0.1 μg/mL of methotrexate, UV + EHMF-L (125 μg/mL) group receiving low-dosage of EHMF, and UV + EHMF-H (500 μg/m) group treated with high-dosage of EHMF.

The high and low doses of EHMF were determined according to the cell survival rate greater than 80% in the pre-experimental results, respectively.

#### 2.4.1 Determination of HaCaT cell cytotoxicity by the CCK-8 assay *in vitro*

HaCaT cells were seeded in 96-well plates at a density of 5×10^3^ cells/well and treated with corresponding drugs for 2 h prior to UV irradiation (UVB 20 mJ/cm^2^ + UVA 200 mJ/cm^2^) for 30 min to establish an *in vitro* model of UV-induced photoaging. Cells in the control group received no treatment. Post-irradiation, CCK-8 reagent was added, and the cells were incubated for 2–4 h. Cell viability was assessed by measuring the optical density (OD) at a wavelength of 450 nm.

#### 2.4.2 Effect of EHMF on HaCaT cell by ELISA assay *in vitro*

HaCaT cells were seeded in 96-well plates at a density of 5×10^3^ cells/well and treated with corresponding drugs for 2 h prior to UV irradiation (UVB 20 mJ/cm^2^ + UVA 200 mJ/cm^2^) for 30 min to establish an *in vitro* model of UV-induced photoaging. Cells in the control group received no treatment. Post-irradiation, the expression levels of IL-6, IL-17, TNF-α, and EGFR in the cell supernatant were quantified using commercially available ELISA kits, following the manufacturer’s protocols. OD values were measured at 450 nm using a microplate reader to determine the concentrations of these markers.

#### 2.4.3 Immunofluorescent staining *in vitro*

HaCaT cells were seeded in 6-well plates at a density of 1×10^5^ cells/well and allowed to adhere and spread. Cells were treated with the respective pharmacological agents for 2 h prior to being exposed to ultraviolet light (UVB 20 mJ/cm^2^ + UVA 200 mJ/cm^2^) for 30 min to establish a model of photoaging. Cells in the control group received no treatment. To differentiate between live and dead cells, Calcein-AM and propidium iodide (PI) were employed for staining, respectively. For fluorescence microscopy analysis, live cells were visualized in yellow-green fluorescence upon excitation at a wavelength of 490 ± 10 nm, followed by the visualization of dead cells in red fluorescence, excited at a wavelength of 545 nm. This dual-staining technique facilitated the assessment of cell viability and integrity within the experimental setup.

### 2.5 Animal experiments verification

#### 2.5.1 Chemicals and reagents

IL-17, IL-6, TNF-α and EGFR Elisa kits (ZCIBIO Technology Co.,Ltd., China). Methotrexate (Shanghai, China). UV lamps (Guangdong, China); BCA Protein Assay Kit (cwbiotech, 02912E), Live/Dead Cell Double Staining Kit (Biogradetech, B-CHK103-500T). Methanol (LC-MS, Merck KGaA), Acetonitrile (LC-MS, Merck KGaA), 2-propanol (LC-MS, Merck KGaA). Acrylamide (Amresco, 0341), Bis-Acrylamide (Amresco, 0172), Glycine (Sigma, G8898), SDS (Sigma, L4390), APS (Amresco, 0486), Trizma base (Sigma, T1503), goat anti-rabbit IgG (Jackson, 111-035–003), goat anti-mouse IgG (Jackson, 115-035–003), nacl (Sinopharm, 10019392), ECL (Millipore, WBKLS0500), Non-fat milk (Erie, yili), Protease inhibitor cocktail (Roche, 04693116001), Tween-20 (Amresco, 0777).

#### 2.5.2 Animals and grouping

Specific pathogen-free (SPF) male ICR mice (SCXK (Xiang) 2022-0010) weighing 20–22 g were obtained from Changsha Tianqin Biotechnology Co., Ltd. The animals were housed in a controlled environment with a temperature of 22 ± 2°C and a 12-h light-dark cycle. All experimental protocols involving animal use were meticulously designed and conducted following ethical guidelines, and approval was obtained from the Animal Experiment Ethics Committee of Guizhou University of Traditional Chinese Medicine (SYXK (Qian) 2021-0005).

The mice were randomly divided into six groups (n = 6): (1) Control, (2) Model group (UV radiation), (3) UV + MTX (10 mg/kg), (4) UV + EHMF-L (200 mg/kg), (5) UV + EHMF-H (400 mg/kg), (6) UV + Gel.

According to the results of the preliminary experiment, the high and low doses of EHMF were determined to be 200 mg/kg and 400 mg/kg, respectively. In order to facilitate transdermal drug delivery, carbomer simple gel was prepared for drug loading to facilitate drug delivery to each treatment group, so UV + Gel group was set up for subsequent efficacy verification.

#### 2.5.3 UV-induced skin photoaging model and experimental design

24 h before the experiment, the dorsal hair of each mouse was carefully shaved, exposing an area of approximately 3 × 3.5 cm^2^. With the exception of the control group, all other groups were placed in a deep box positioned directly below the phototherapy apparatus for UV radiation exposure. Based on previous literature (Katayoshi et al., 2021; Sun et al., 2024) and the results of preliminary experiments, the UV modeling conditions were established and confirmed. The UV radiation dose was set at UVB 20 mJ/cm^2^ + UVA 200 mJ/cm^2^ for 48 h.

The drugs were topically applied to the skin of the respective treatment groups, while the positive control group received a topical application of MTX once daily for seven consecutive days. The UV + Gel group was applied an appropriate amount of carbomer gel. The control and model groups were not treated.

#### 2.5.4 Skin apparent evaluation

After establishing the UV-induced skin photoaging model, the dorsal skin of the mice displayed characteristic signs, such as localized thickening, laxity, prominent deep wrinkles, and hyperpigmentation, confirming successful model construction. The dorsal skin condition of each mouse was observed daily before drug administration. On day 7, 1 hour post-administration, photographs of the dorsal skin were taken, and the skin condition was evaluated using a three-person blind method. Apparent scores were assigned according to the skin wrinkle rating scale (Table 1), and the average scores were used for analysis.

**TABLE 1:** Skin wrinkle rating scale.

Score	Degree	Appearance of skin
0	Normal Skin	Fine skin lines, no wrinkles
1	Mild Wrinkles	A few superficial transverse wrinkles
2	Moderate Wrinkles	Superficial wrinkles have completely disappeared, leaving severe and persistent deep wrinkles
3	Severe Wrinkles	Superficial wrinkles disappeared completely, while deep wrinkles are severe and persistent
4	Very Severe Wrinkles	Deep wrinkles are severe and persistent, accompanied by skin flushing

#### 2.5.5 Histological analysis *in vivo*

After the seventh day of treatment, the mice were euthanized by cervical dislocation. The dorsal skin tissue was meticulously dissected and promptly fixed in 4% paraformaldehyde (v/v) for 48 h. The specimens then underwent a series of processing steps, including dehydration, paraffin embedding, sectioning, and dewaxing. Finally, hematoxylin and eosin (HE) staining and Masson staining were performed to assess the histopathological changes in the tissue.

#### 2.5.6 Quantification of inflammatory cytokines by ELISA assay *in vivo*

The expression of TNF-α, IL-6, IL-17, and EGFR in mice skin tissue was assessed using an Elisa method at a wavelength of 450nm, as instructed by the manufacturer.

#### 2.5.7 Immunohistochemistry assay *in vivo*

Paraffin-embedded skin tissue sections were routinely dewaxed and incubated with 3% H₂O₂ in deionized water at room temperature for 25 min to eliminate endogenous peroxidase activity. After rinsing three times with PBS (5 min each), sections were immersed in a citric acid antigen retrieval solution and heated to boiling in a microwave oven. The antigen retrieval process was repeated twice at 8-min intervals. After cooling, sections were incubated with goat serum blocking solution at 37°C for 30 min, then shaken dry, and incubated overnight at 4°C with appropriately diluted primary antibodies. After three washes with PBS (5 min each), biotin-labeled goat anti-rabbit IgG was added and incubated at 37°C for 50 min. Sections were then rinsed three times with PBS (5 min each), incubated with the secondary antibody at 37°C for 30 min, and washed again. The reaction was monitored under a microscope using DAB as a chromogenic agent, followed by rinsing with tap water and counterstaining with hematoxylin for 3 min. Finally, the sections were dehydrated and sealed using transparent neutral gum. Quantitative analysis was performed using Image-Pro Plus 6.0 software under standardized light intensity conditions.

#### 2.5.8 Western blot *in vivo*

Proteins were extracted from homogenized mice skin tissue on ice. Following electrophoresis on an SDS-polyacrylamide gel, the proteins were transferred onto a PVDF membrane and blocked with 5% BSA for 1 h. The membrane was then incubated overnight at 4°C with primary antibodies against AKT (1:1000), P-AKT (1:1000), STAT3 (1:1000), P-STAT3 (1:1000), and GAPDH (1:1000). After incubation with a horseradish peroxidase-labeled secondary antibody (1:1000) for 40 min at room temperature, the proteins were visualized using an ECL detection kit and imaged with a gel imaging system. Image analysis was performed using ImageJ software to quantify the grayscale values.

### 2.6 Statistical analysis

Prism 8 (GraphPad Software, USA) was used for all analyses. All data are presented as mean ± standard deviation. Differences between groups were analyzed by unpaired Student’s t-test or one-way analysis of variance (ANOVA) followed by Dunnett’s multiple comparison test. *p* < 0.05 was considered statistically significant.

## 3 Results

### 3.1 Analysis of components of the EHMF

Using UPLC-MS/MS, we generated detailed total ion current profiles of EHMF, displaying abundant chromatographic peaks in both ion modes. Comparative analysis with our proprietary mass spectrometry library allowed the identification of 22 active compounds, each exhibiting a drug similarity index no less than 0.18 (DL ≥ 0.18). Further analysis revealed 22 major chromatographic peaks, precisely corresponding to specific bioactive compounds from EHMF, as shown in Table 2. This robust methodology not only confirms the presence of these bioactive agents but also underscores their significant roles in distinguishing between group differences, demonstrating the precision and reliability of our analytical approach in the identification of botanical extracts’ constituents.

**TABLE 2:** The main active compounds of EHMF.

Peak No.	Compound	Formula	RT (min)	Calc. MW
1	Kaempferol	C_15_H_10_O_6_	9.863	286.04735
2	Morin	C_15_H_10_O_7_	6.426	302.04201
3	Naringenin	C_15_H_12_O_5_	6.574	272.06804
4	Sterigmatocystin	C_18_H_12_O_6_	5.292	324.06301
5	Isorhamnetin	C_16_H_12_O_7_	7.147	316.05776
6	Methysticin	C_15_H_14_O_5_	9.171	274.0838
7	Naringenin chalcone	C_15_H_12_O_5_	7.688	272.06819
8	Artemisinin	C_15_H_22_O_5_	9.222	282.14629
9	Scrophulein	C_17_H_14_O_6_	11.569	314.07845
10	Artemotil	C_17_H_28_O_5_	11.023	312.19301
11	Dihydroartemisinin	C_15_H_24_O_5_	9.947	284.16194
12	Quercetin	C_15_H_10_O_7_	5.876	302.04222
13	Muscone	C_16_H_30_O	17.037	238.22927
14	5-O-methyl embelin	C_18_H_28_O_4_	11.143	308.19821
15	Phloionolic acid	C_18_H_36_O_5_	11.211	332.25563
16	Kaempferol 3-glucorhamnoside	C_27_H_30_O_15_	6.767	594.15793
17	Linarin	C_28_H_32_O_14_	8.063	592.17894
18	Linderane	C_15_H_16_O_4_	10.135	260.10451
19	Methyl 4-hydroxy-3-methoxycinnamate	C_11_H_12_O_4_	7.585	208.07344
20	Isosteviol	C_20_H_30_O_3_	12.781	318.21889
21	4′,7-Di-O-methylnaringenin	C_17_H_16_O_5_	9.613	300.09935
22	Clareolide	C_16_H_26_O_2_	11.956	250.19292

### 3.2 Mining of core targets of EHMF

**FIGURE 1:**
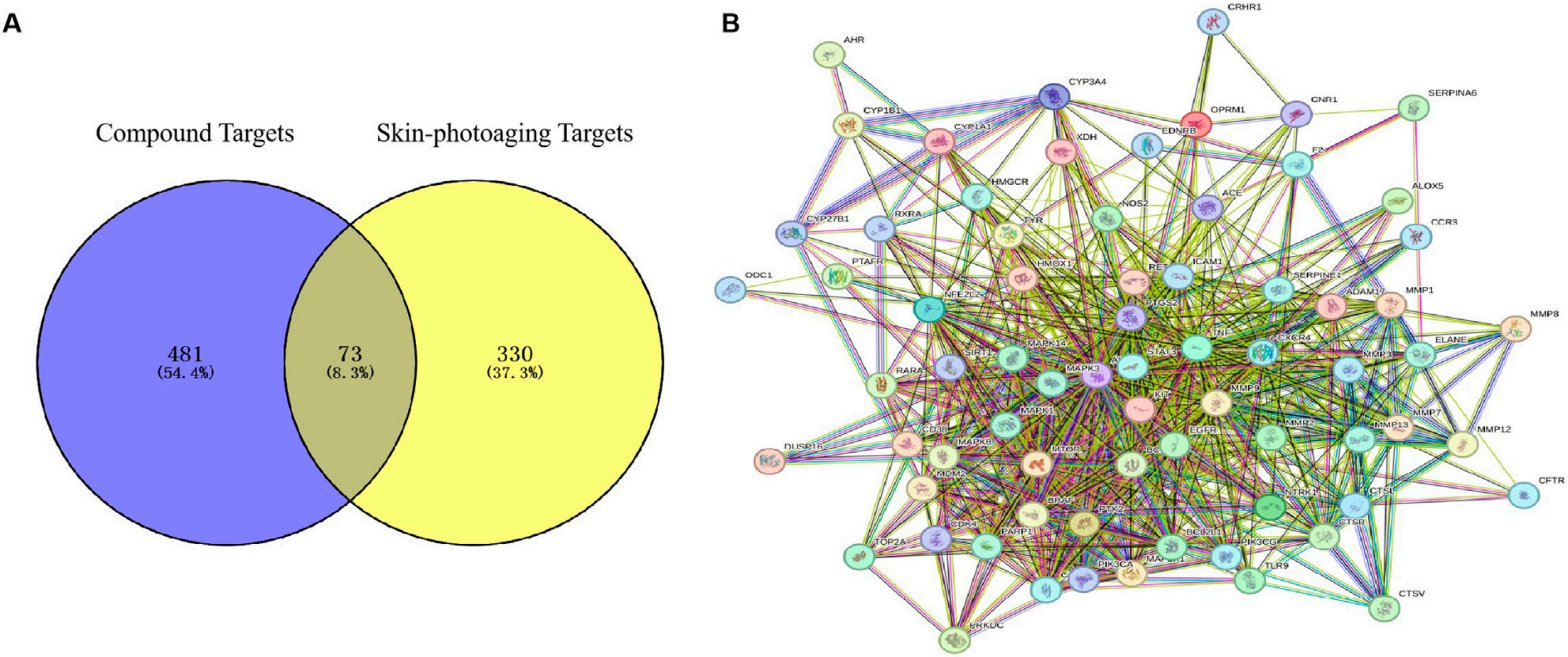
**(A)** The EHMF compound targets, skin photoaging disease targets and the overlapping targets by Venn diagram. **(B)** The PPI network of intersection targets between compound and UV-induced skin phtoaging disease-related targets.

**FIGURE 2:**
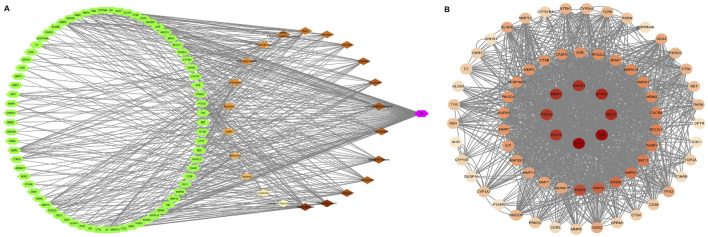
**(A)** “herb-compound-target” network: EHMF compound targets, skin photoaging disease and targets network. **(B)** Key targets of compound and UV-induced skin phtoaging disease-related targets analysis results.

Using the GeneCards database, we identified 403 targets associated with UV-induced skin photoaging. A Venn diagram (Figure 1A) highlights 73 targets at the intersection of EHMF and skin photoaging, indicating their potential relevance. The network of active ingredients and these targets was constructed using Cytoscape 3.8.2, with a statistical analysis of the degree values of each node. To further elucidate and predict the central core targets of EHMF, a PPI network was developed using these intersecting targets, facilitated by the STRING database (Figure 1B). The five most prominent active compounds, exhibiting the highest network degrees, were identified as Dihydroartemisinin, 4’,7-Di-O-methylnaringeni, Scrophulein, Kaempferol, and Quercetin. Similarly, the top five targets with the highest degrees, playing crucial roles in skin photoaging pathogenesis, were AKT1, TNF-α, STAT3, MMP9, and EGFR (Figures 2A, B). This analysis provides a deeper understanding of the molecular interactions underpinning the therapeutic potential of EHMF against skin photoaging.

### 3.3 Enrichment analysis of EHMF

**FIGURE 3:**
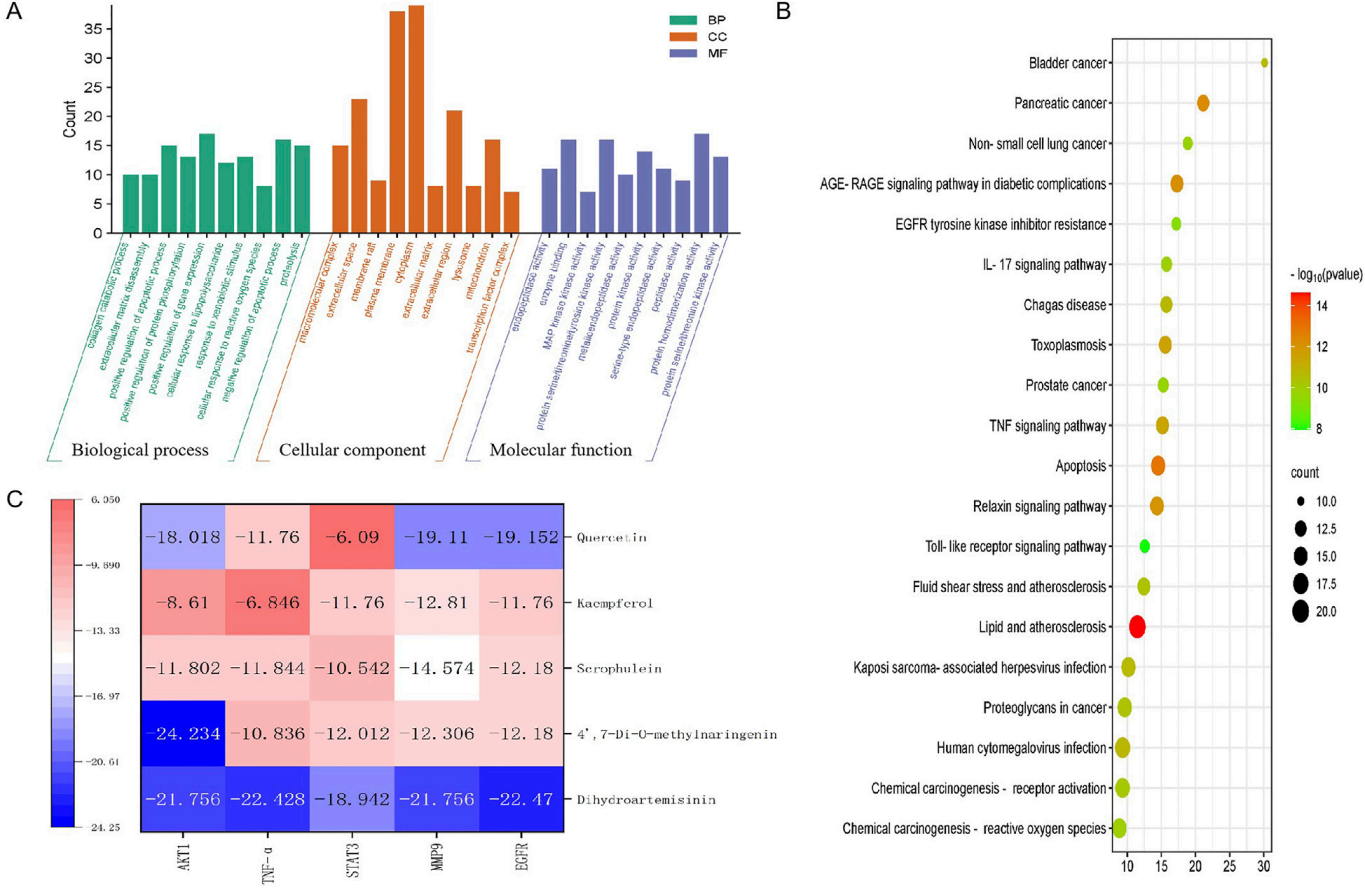
**(A)** GO analysis results. **(B)** KEGG pathway enrichment results. **(C)** Molecular docking thermogram between the top 5 active components and the top 5 intersection targets according to the score of Degree.

To elucidate the roles of intersecting target proteins in gene functions and signaling pathways, both GO enrichment analysis and KEGG pathway analysis were conducted using the Metascape database, with results visualized for clarity. The GO enrichment analysis yielded 407 biological processes (BP), 46 cellular components (CC), and 74 molecular functions (MF). The top ten results of this analysis are displayed in Figure 3A. Notably, the most significant BP included the positive regulation of gene expression, positive regulation of transcription from the RNA polymerase II promoter, and the negative regulation of the apoptotic process. The CC results indicated that the impact of EHMF on skin disease predominantly occurs within the cytoplasm, plasma membrane, and cytosol. MF critical to the alleviation of skin photoaging symptoms, such as protein binding, identical protein binding, and ATP binding, were identified. Furthermore, KEGG analysis revealed that approximately 30 pathways were enriched, as shown in Figure 3B. Additionally, the signaling pathways and associated targets were integrated into a “target-signaling pathway” network, constructed using the R programming language.

### 3.4 Docking analysis predicted the potential binding activities of compounds and targets

**FIGURE 4:**
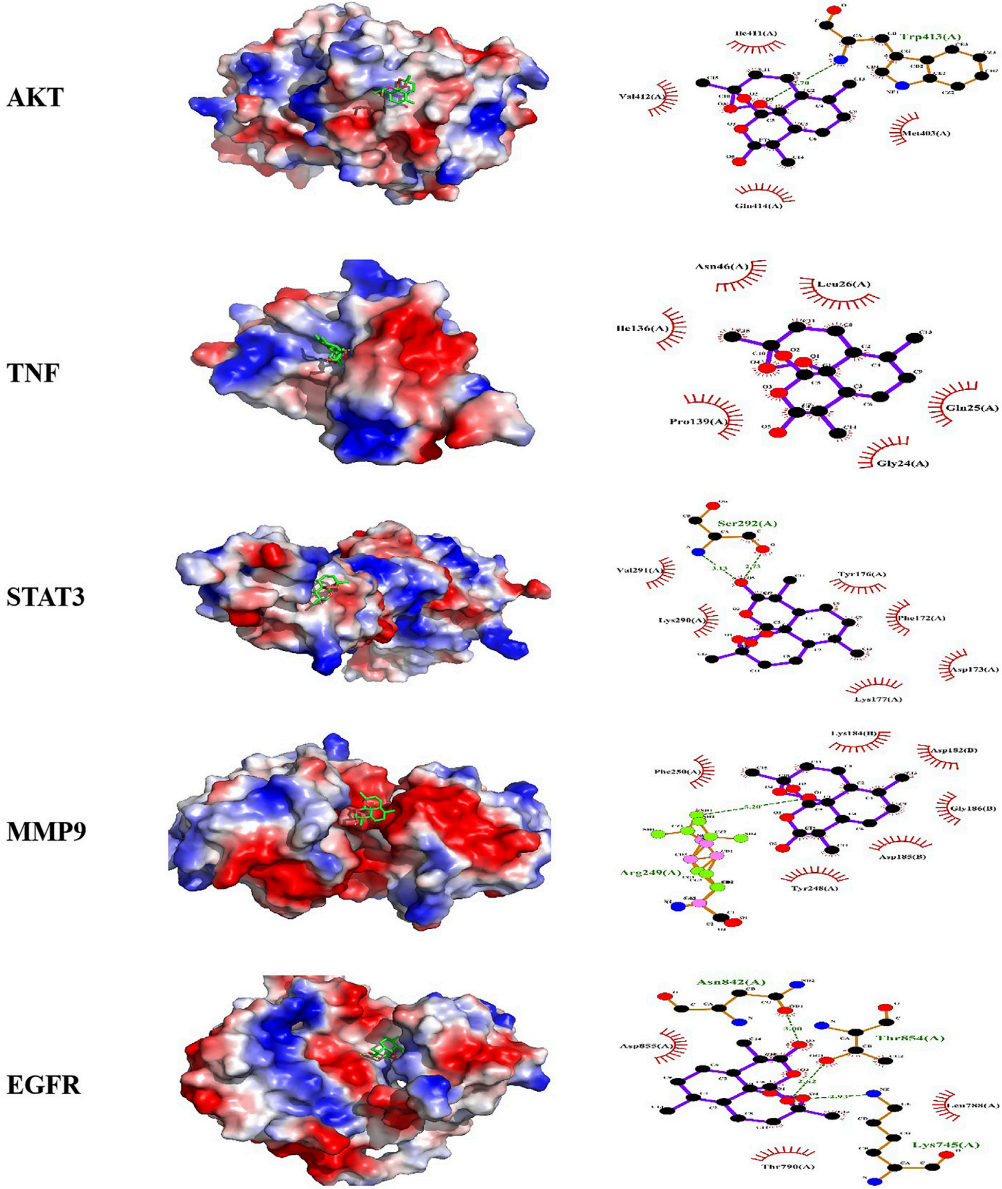
Molecular docking models of active compound binding to potential targets. Molecular docking pattern diagram of AKT1, TNF-α, STAT3, MMP3, EGFR.

Heatmaps of docking scores for Scrophulein, Quercetin, Kaempferol, 4′,7-Di-O-methylnaringenin, and Dihydroartemisinin with the top five core targets are presented in Figure 3C. As indicated by it, Dihydroartemisinin achieved the highest docking scores with the top five core targets. The three-dimensional and two-dimensional binding modes of Dihydroartemisinin with each protein are shown in Figure 4. Specifically, the binding of Dihydroartemisinin to AKT1 involves residues ILe411, Val412, Gln414, Met403, and Trp413, with Dihydroartemisinin forming a hydrogen bond with Trp413’s O1. The interaction with EGFR is mediated through strong hydrogen bonds formed by Asn842, Thr854, and Lys745 with Dihydroartemisinin. For MMP9, the binding pocket residues Phe250, Arg249, Lys184, Asp182, Gly186, Asp185, Tyr248, and Tyr248 are involved in the binding, with a hydrogen bond forming between Dihydroartemisinin and Arg249. The interaction with STAT3 is facilitated by Val291, Lys290, Kys177, Asp173, Phe172, Tyr176, and Ser292, with Ser292 forming dual hydrogen bonds with Dihydroartemisinin. Lastly, TNF-α primarily interacts with Dihydroartemisinin through hydrophobic forces involving Ile136, Pro139, Gly24, Gln25, Leu26, and Asn46.

### 3.5 Concentration-dependent protection of EHMF against HaCaT cells and reduction of UV-induced damage

**FIGURE 5:**
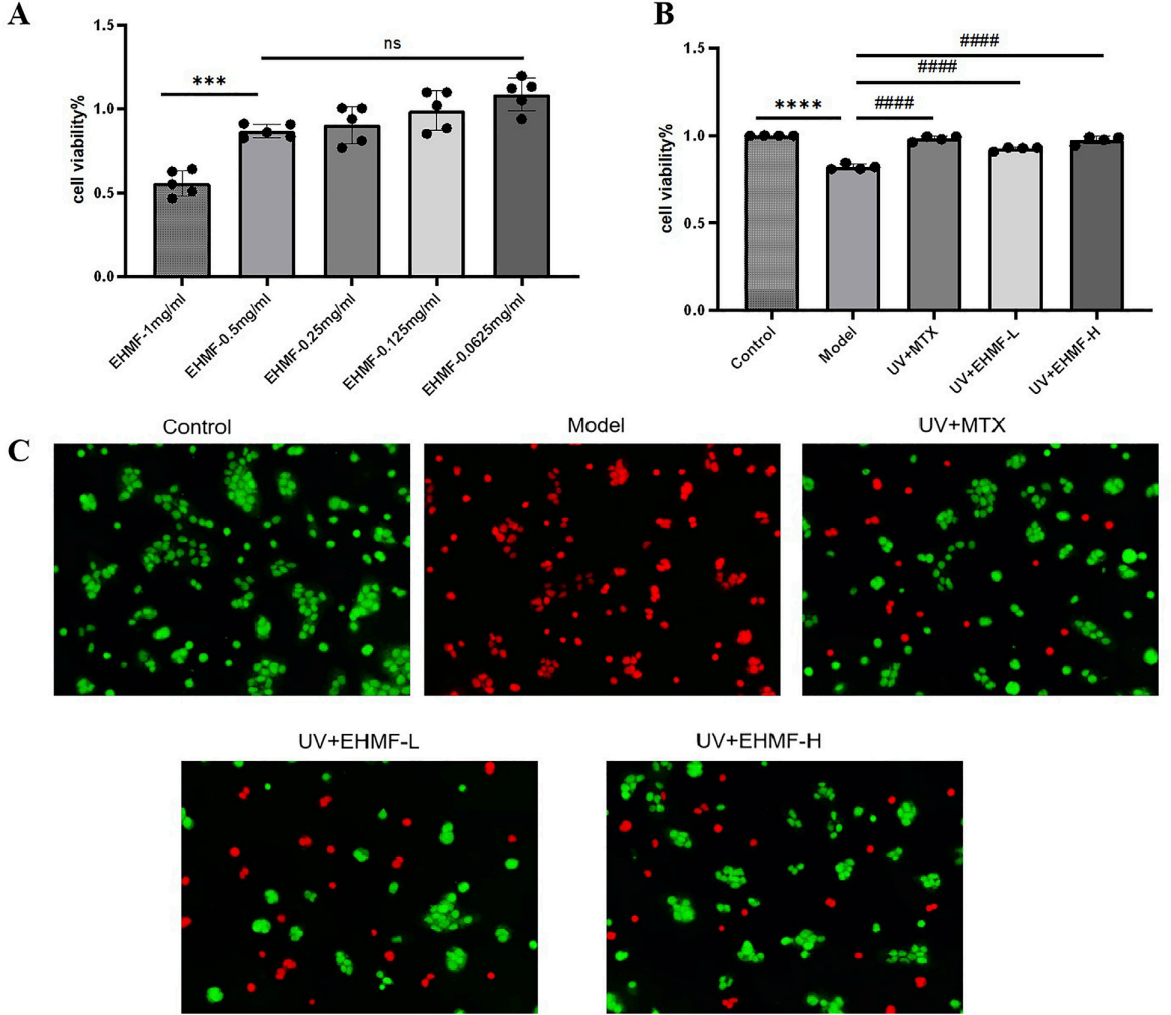
Effect of EHMF on HaCaT cell viability and protective efficacy against UV-induced skin photoaging. **(A)** The effect of different dosage of EHMF on the viability of HaCaT cells. **(B)** The effect of different dosage of EHMF on the viability of HaCaT cells after UVB 20 mJ/cm2 + UVA 200 mJ/cm2 for 30 minutes. **(C)** Staining of different groups of live/dead cells after UV radiation. (*p < 0.05, **p < 0.01, ***p < 0.001, ****p < 0.0001 compared with the Control group. #p < 0.05, ##p < 0.01, ###p < 0.001, #### < 0.0001 comparison with the Model group. ns, no significant).

The effect of EHMF on the viability of HaCaT cells was illustrated in Figure 5A. Contrary to a dose-dependent proliferative effect, the results demonstrated a significant reduction in cell viability at the highest concentration tested (1 mg/mL), as indicated by the statistically significant decrease (*p* < 0.001) compared to lower concentrations. Notably, there was no significant difference in cell viability between the concentrations of 0.5 mg/mL, 0.25 mg/mL, and 0.125 mg/mL (ns, non-significant), indicating that reductions in concentration below 0.5 mg/mL do not further increase cell survival. These findings suggest that while EHMF at 1 mg/mL exhibits cytotoxic effects, lower concentrations are well-tolerated by HaCaT cells. Consequently, for subsequent experiments, concentrations of 0.5 mg/mL and 0.125 mg/mL were selected to represent high and low dose treatments, respectively.

After UV irradiation, the survival rate of HaCaT cells in the model group was significantly lower compared to that in the control, unirradiated control group (*p* < 0.0001). However, cells treated with both low and high concentrations of EHMF (EHMF-L and EHMF-H) maintained cell viability at levels comparable to those observed in the UV + MTX group, with no significant differences between them (*p* > 0.05), demonstrating a robust protective effect against UV damage. Notably, cell viability in the EHMF-treated groups was significantly higher than that in the model group (*p* < 0.001), indicating that EHMF effectively counteracts UV-induced cellular damage, potentially through anti-inflammatory and reparative mechanisms. These results suggest that EHMF could be a valuable dermatological agent, particularly for mitigating the effects of UV exposure on skin cells, as further detailed in Figure 5B.

Further confirming its protective efficacy, EHMF demonstrated significant preservation of cell viability under UV stress conditions, as depicted in Figure 5C. The model group, which received no protective treatment, displayed a substantial increase in red staining, indicative of high cell mortality due to UV exposure. In contrast, both the UV + MTX group, serving as a positive control, and the UV + EHMF-H group exhibited significantly improved cell survival, with approximately 80% of cells appearing green, indicative of effective protection against UV-induced damage. The morphology of cells in these groups remained predominantly normal, with an estimated 20% mortality. Conversely, the UV + EHMF-L group displayed about 60% cell survival, suggesting that while the lower concentration of EHMF does offer some protective benefits, it is less effective than the higher concentration. These findings affirm that EHMF, particularly at higher concentrations, provides substantial protective effects against UV radiation, akin to those of the established MTX treatment in HaCaT cells.

### 3.6 EHMF effectively reduces inflammation and regulates EGFR *in vitro* and *in vivo*

**FIGURE 6:**
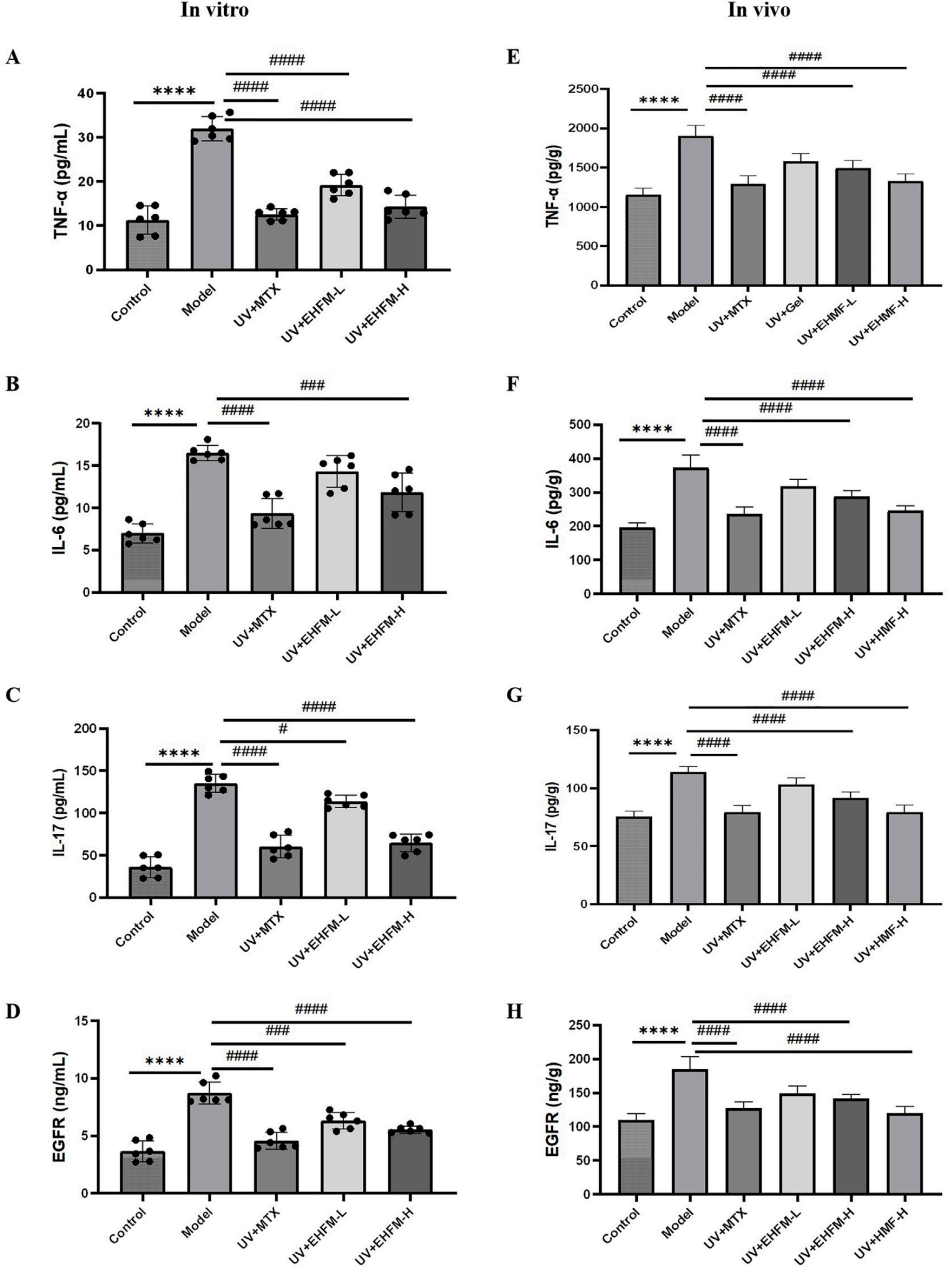
Effects of low and high dosage of EHMF on inflammatory cytokine and EGFR expression in UV-induced HaCaT cell in vitro treated with corresponding drugs for 2 hours prior to UV irradiation (UVB 20 mJ/cm2 + UVA 200 mJ/cm2) for 30 minutes and skin photoaging-mice in vivo treated with corresponding drugs for 2 hours prior to UV irradiation (UVB 20 mJ/cm2 + UVA 200 mJ/cm2) for 48 hours. The levels of TNF-α **(A)**, IL-6 **(B)**, IL-17 **(C)**, EGFR **(D)** in HaCaT cell by ELISA kits. Secretion levels of TNF- a **(E)**, IL-6 **(F)**, IL-17 **(G)**, EGFR **(H)** in mice skin tissue. (*p < 0.05, **p < 0.01, ***p < 0.001, *p <0.0001 compared with the Control group. #p < 0.05, #p < 0.01, ###p < 0.001, ####p <0.0001 comparison with the Model group).

EHMF treatment significantly reduced IL-6, TNF-α, and IL-17 levels in the HaCaT skin photoaging cell model, effectively decreasing skin inflammation (Figures 6A–C). The EHMF-H group particularly enhanced EGFR expression (*p* < 0.01), reinforcing EHMF’s role in enhancing the skin’s anti-inflammatory response (Figure 6D). These results demonstrate EHMF’s potential therapeutic benefits in combatting photoaging by modulating pro-inflammatory cytokines and EGFR expression.

Subsequently, animal experiments further validated these findings. Excessive UV irradiation markedly increases the expression of inflammatory markers such as IL-6, IL-17, and TNF-α in skin epidermal and dermal cells, leading to inflammation and associated skin disorders. Figures 6E–G illustrated significant cytokine elevation in the UV-exposed model group compared to the control control, confirming UV’s role in exacerbating skin inflammation. After 7 days of treatment, significant reductions in IL-6, IL-1β, and TNF-α were observed in both the MTX and EHMF-treated groups, with the TNF-α reduction notably pronounced in the EHMF groups. Additionally, EGFR levels, crucial for maintaining skin homeostasis and abnormally increased by UV exposure, were effectively normalized in the treatment groups (Figure 6H). These results underscore EHMF’s efficacy in mitigating UV-induced increases in inflammatory markers and overexpression of EGFR, further substantiating its potential for treating UV-related skin inflammation.

### 3.7 EHMF treatment preserves skin integrity by reducing inflammatory and skin photoaging markers *in vivo*

**FIGURE 7:**
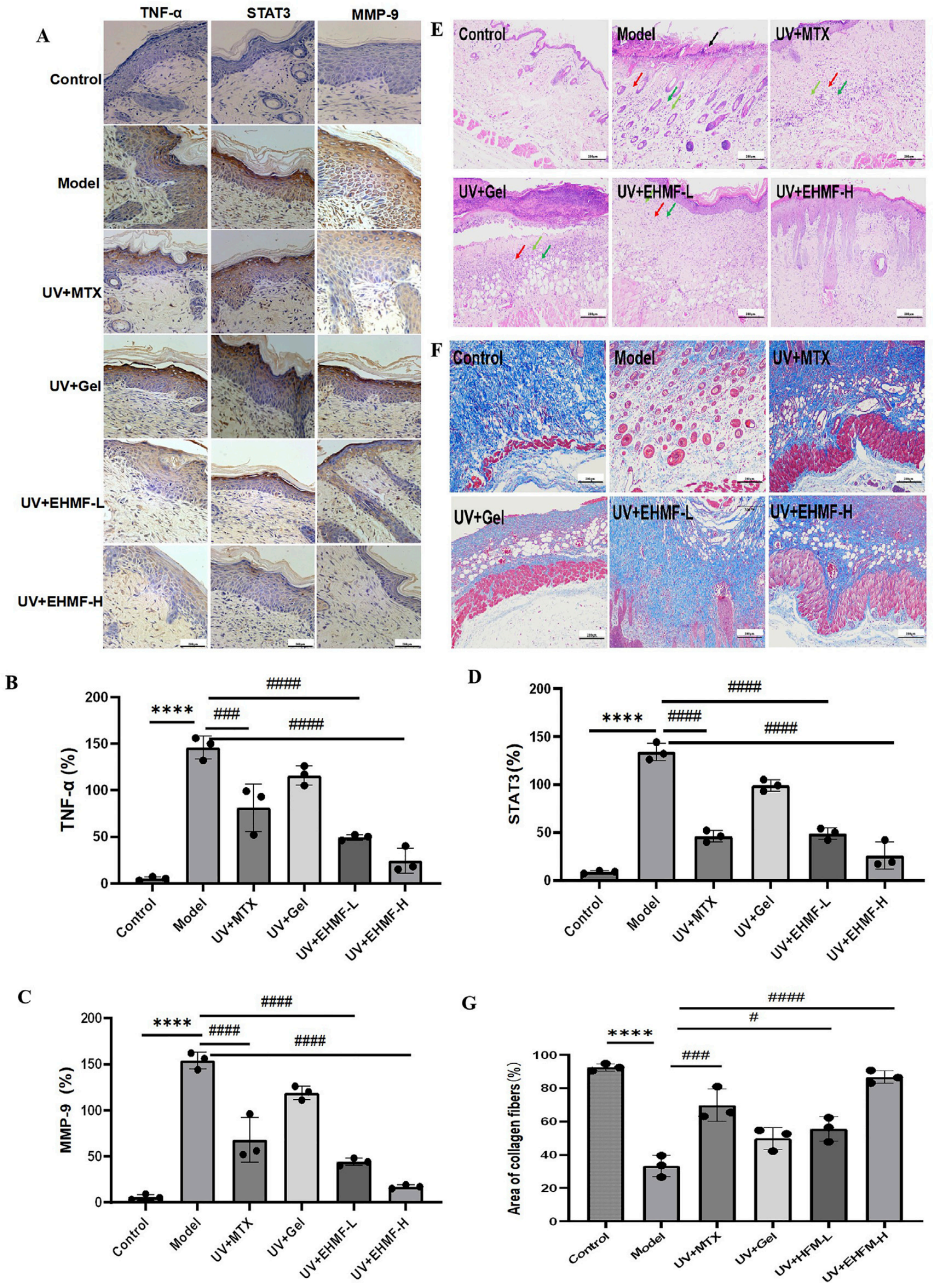
Immunohistochemical analysis and Histopathological evaluation of UV-induced skin photoaging and the protective effects of EHMF. **(A)** Immunohistochemical pictures of TNF-a, MMP-9 and STAT3 in different groups of mice. **(B–D)** Secretion levels of TNF-α **(B)**, MMP-9 **(C)**, STAT3 **(D)** in mice skin tissue. The skin tissue were stained with H&E **(E)** and Masson’s trichrome staining **(F)**.The area ratio of collagen fibers in different groups of mice **(G)**. Scale bars are 200 um. (*p < 0.05, **p < 0.01, ***p < 0.001, ****p < 0.0001 compared with the Control group. #p < 0.05, ##p < 0.01, ###p < 0.001, ####p < 0.0001 comparison with the Model group).

Immunohistochemical analysis demonstrates significant upregulation of MMP-9 in the model group, disrupting the extracellular matrix and inducing skin photoaging symptoms such as skin laxity and wrinkles (Figure 7A). Treatment with EHMF significantly mitigates this upregulation in a concentration-dependent manner, with all differences statistically significant (Figures 7B–D). Furthermore, STAT3 and TNF-α, essential for keratinocyte differentiation and skin integrity, also show marked increases in the model group. EHMF treatment effectively reduces the expression of these key regulators, confirming its potential to modulate inflammatory signaling pathways crucial for maintaining skin health.

H&E and Masson’s trichrome staining were employed to evaluate epidermal thickness and collagen density and arrangement in the dorsal skin of mice. As illustrated in Figure 7E, epidermal thickness in the model group was significantly increased compared to the control group, exhibiting both increased thickness and disorganized structure. Treatment with EHFM-L and EHFM-H resulted in a notable improvement, with epidermal thickness approaching that of the control group. Figure 7F reveals that in the control group, collagen fibers in the dermis were densely packed and well-organized, appearing as extensive blue areas. In contrast, the model group showed a significant reduction in collagen content with disorganized and erratically aligned fibers. Compared to the model group, both EHFM-L and EHFM-H treatments markedly increased collagen density and improved fiber arrangement, with the most pronounced enhancement observed in the EHFM-H group (Figure 7G). These findings demonstrate that EHFM effectively mitigates UV-induced alterations, significantly reducing epidermal thickening and reversing collagen degradation.

### 3.8 EHMF significantly reduce UV-induced skin wrinkles and roughness *in vivo*

**FIGURE 8:**
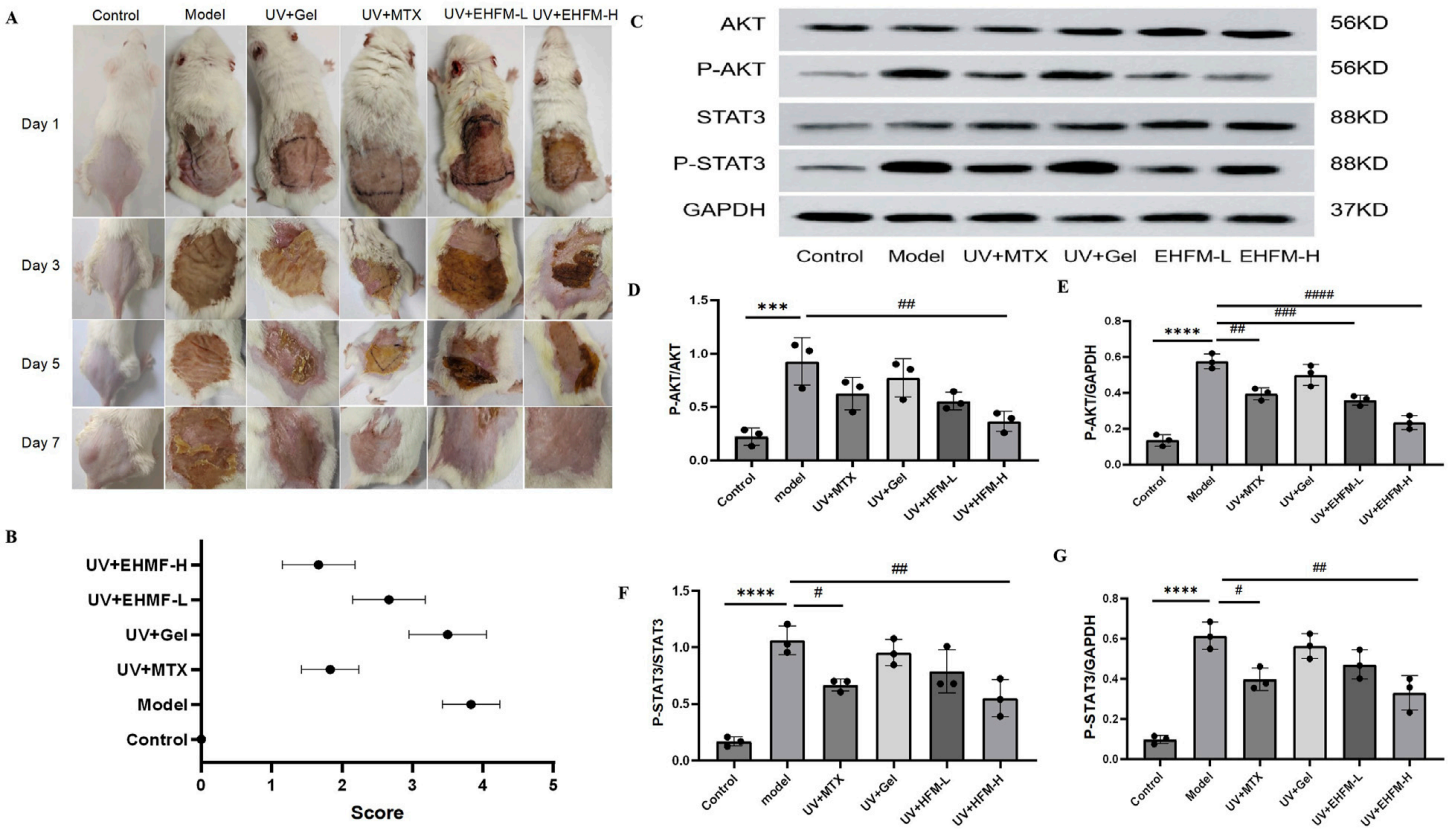
**(A)** Effects of EHFM on the appearance of the UV-induced skin photoaging in mice. **(B)** Apparent scores were assigned according to the skin wrinkle rating scale. **(C–G)** The protein blot images **(C)** and relative expression levels of P-AKT/AKT **(D)**, P-AKT/GAPDH **(E)**, P-STAT3/STAT3 **(F)**, P-STAT3/GAPDH **(G)**. Protein expression levels were quantified by ImageJ software. (*p < 0.05, **p < 0.01, ***p < 0.001, *p < 0.0001 compared with the Control group. #p < 0.05, #p < 0.01, ###p < 0.001, ####p < 0.0001 comparison with the Model group).

The investigation delineated notable differences in the dermatological response to various treatments in an aged mice model (Figures 8A, B). In the control group, characterized by untreated mice, the skin was smooth and thin with clearly visible, flesh-colored subcutaneous blood vessels, indicative of a healthy baseline. Conversely, the model group, subjected to UV radiation, exhibited significant dermatological deterioration, marked by deep wrinkles, pronounced hypertrophy, and redness, establishing a robust model for skin photoaging. Treatment with a carbomer-based gel in the blank gel group improved skin conditions compared to the model group, yet slight roughness and redness remained. This outcome suggests that while carbomer has moisturizing capabilities, it only partially mitigates the effects of skin photoaging. Notably, the application of MTX resulted in substantial improvements, reducing skin wrinkles significantly. This effect can be attributed to MTX’s anti-inflammatory properties and its role in shielding skin cells from oxidative damage. Moreover, EHMF-H group displayed considerable improvements in both skin texture and wrinkle severity, with the skin becoming significantly thinner and only minimally reddened. In stark contrast, EHMF-L treatment failed to produce similar results; the skin remained rough, and symptoms such as redness and scabbing were prevalent. These findings clearly demonstrate the potential of methotrexate and EHMF-H as effective interventions for reversing the signs of skin aging and enhancing dermal health in aged mice. The differential responses also underscore the dose-dependent efficacy of EHMF in treating age-related skin changes.

### 3.9 EHMF significantly modulates AKT and STAT3 phosphorylation in a dose-dependent manner in skin photoaging

To explore the molecular underpinnings by which EHMF ameliorates skin photoaging, we analyzed the expression and phosphorylation profiles of critical proteins within the AKT and STAT3 signaling cascades in mice afflicted with skin photoaging. Through KEGG pathway analysis, the AKT-STAT3 axis emerged as a pivotal therapeutic conduit, integral to the modulation of inflammatory responses. Figure 8C illustrates that EHMF treatment significantly attenuated the hyperphosphorylation of AKT and STAT3 proteins, typically elevated in the skin tissues of skin photoaging mice.

Relative to the baseline established by the control group, the model group demonstrated a marked increase in the phosphorylated forms of AKT (P-AKT) and STAT3 (P-STAT3) in skin tissues (*p* < 0.05). Conversely, EHMF treatment resulted in a dose-dependent decrease in these phosphorylation levels when compared to the model group, with each inter group comparison revealing statistically significant differences (*p* < 0.05) as shown in Figures 8D–G. It is noteworthy that the phosphorylation levels in EHMF-H group were comparable to those in the MTX group, with no significant differences observed (*p* > 0.05).

These findings underscore the multifaceted nature of EHMF’s action in treating skin photoaging, suggesting involvement of multiple biochemical pathways. This data corroborates the notion that TCM may confer therapeutic effects through the coordinated modulation of multiple signaling pathways and targets, illustrating the complexity and potential synergistic interactions within its mechanism of action.

## 4 Discussion

In this study, an integrated strategy combining network pharmacology and UPLC-MS/MS was employed to elucidate the active ingredients of EHMF that could effectively alleviate UV-induced skin photoaging. Furthermore, our experimental results clarified the pharmacodynamic mechanism of EHMF in treating skin photoaging and identified its potential pathways of action. We identified a total of 22 active ingredients from HMF, along with 73 cross-targets linked to both HMF and UV-induced skin photoaging. GO enrichment and KEGG pathway analyses emphasized the significance of inflammatory signaling pathways. *In vitro* studies demonstrated that HMF extracts effectively reduced UV-induced inflammatory factors in HaCaT cells and enhanced cell survival rates. *In vivo*, EHMF significantly mitigated skin lesions on the backs of UV-exposed mice, resulting in decreased epidermal and dermal thickening and reduced pathological infiltration of inflammatory cells. Additionally, it lowered abnormal MMP-9 expression and collagen fiber proliferation, as well as the levels of inflammatory factors such as TNF-α, IL-6, IL-17, and EGFR. Results from Western blotting and immunohistochemistry confirmed that the over-activation of the AKT-STAT3 signaling pathway was inhibited.

Network pharmacology identified 403 potential targets responsible for UV-induced skin photoaging, while 73 components were related to EHMF. Experimental validation confirmed five core targets obtained through KEGG and GO analyses: AKT1, TNFα, STAT3, MMP9, and EGFR. EHMF exhibited potent anti-inflammatory effects, notably reducing the expression of pro-inflammatory cytokines like TNF-α, IL-6, and EGFR, which are typically upregulated in response to UV exposure and play a crucial role in accelerating skin aging and exacerbating inflammatory responses (Wang et al., 2014). Skin photoaging is known to induce an elevation of inflammatory factors such as TNF-α, IL-1β, and IL-6 (Huang et al., 2024; Zhang et al., 2023). One effective treatment approach for skin photoaging involves alleviating inflammatory markers on the skin surface, regulating the microenvironment, and ultimately relieving skin photoaging symptoms (Yang et al., 2024). In our study, EHMF treatment significantly reduced IL-6, TNF-α, and IL-17 levels in the HaCaT skin photoaging cell model while increasing EGFR expression. The efficacy of EHMF in reducing skin photoaging markers mirrored the effects observed with MTX, suggesting that EHMF could be a viable natural alternative for treating skin photoaging. This potential was further substantiated through *in vivo* experiments in mice and *in vitro* studies using HaCaT cells, where EHMF (0.125–0.5 mg/mL) effectively prevented UV-induced cell death and demonstrated anti-inflammatory properties.

UV radiation, a recognized carcinogen, plays a significant role in skin aging and disorders, where UV radiation promotes the secretion of MMPs, crucial for dermal collagen degradation and leading to the structural collapse of the extracellular matrix (Freitas-Rodríguez et al., 2017). The matrix degradation pathway, particularly through the synergistic action of various MMPs, has increasingly attracted research focus. MMPs are zinc-centered endopeptidases that play a pivotal role in photoaging. UV light can cause a substantial increase in MMP expression in skin fibroblasts by stimulating the synthesis of ROS (Herrling et al., 2006), leading to extensive collagen and elastic fiber degradation (Zague et al., 2018).

Our study utilized LED lamp beads as a UV light source to establish an acute skin photoaging model in mice, simulating the clinical manifestations of mice photodamage disorders. This model confirmed the efficacy of EHMF in reducing signs of aging, such as spots, wrinkles, and inflammatory infiltration. Our findings revealed that EHMF significantly curtailed the activity of MMPs, particularly MMP-9, which is primarily involved in the degradation of type I collagen and contributes to skin photoaging. The overexpression of MMP-9 induced by UV exposure leads to substantial collagen breakdown, a marker of skin photoaging (Cancemi et al., 2020; Geng et al., 2021). By inhibiting these enzymes, EHMF preserves the structural integrity of the dermal collagen matrix, underscoring its potential as a therapeutic agent against UV-induced damage.

The phosphatidylinositol 3-kinase (PI3K)/AKT signaling pathway plays a pivotal role in regulating critical cellular functions such as proliferation, differentiation, migration, angiogenesis, and metabolism (Teng et al., 2021). This pathway’s significance extends beyond normal physiological processes; its hyperactivation is often linked to various pathological conditions, including inflammatory responses and cellular alterations that are characteristic of skin photoaging. Specifically, the dysregulation of this pathway can result in increased apoptosis, heightened oxidative stress, and accelerated collagen degradation, all of which contribute to the adverse effects of UV exposure on skin health (Anik et al., 2022).

Our study’s findings provide compelling evidence that EHMF treatment effectively modulates the PI3K/AKT pathway. We observed a significant reduction in the expression of pro-inflammatory cytokines and inhibition of matrix metalloproteinases (MMPs), with particular emphasis on MMP-9. This enzyme has been closely linked to collagen degradation and the processes underlying photoaging (Li et al., 2024) By suppressing MMP-9 activity, EHMF not only curtails collagen breakdown but also stabilizes the extracellular matrix, thus preserving the structural integrity of the dermal collagen matrix. This is crucial for maintaining skin elasticity and resilience, which are often compromised in photoaged skin. Moreover, our results indicate that EHMF possesses marked anti-inflammatory properties. We noted a substantial reduction in the levels of inflammatory cytokines such as TNF-α, IL-6, and IL-17. Concurrently, EHMF treatment enhanced epidermal growth factor receptor (EGFR) expression in the HaCaT photoaging cell model. EGFR plays a vital role in skin repair and regeneration, and its upregulation can promote cellular proliferation and differentiation, counteracting some effects of photoaging.

Importantly, the PI3K/AKT pathway is also integral to the regulation of autophagy (Hoxhaj and Manning, 2020; Kma and Baruah, 2022), a process that is essential for cellular homeostasis and response to stress. Future studies could delve deeper into the possibility that EHMF’s modulation of AKT-STAT3 signaling not only affects inflammatory responses but may also influence autophagic processes in the context of skin photoaging treatment. Understanding how EHMF interacts with these pathways could provide insights into its wider therapeutic potential. The noted attenuation of AKT and STAT3 phosphorylation by EHMF further underscores its promising role in mitigating other inflammatory skin disorders where these signaling pathways are implicated. By addressing both inflammation and the structural integrity of the skin, EHMF may represent a multifaceted approach to combatting the effects of UV damage and promoting healthier skin. Overall, these findings advocate for the importance of further investigation into EHMF’s mechanisms of action and its potential applications in dermatological treatments, potentially leading to more effective strategies for managing skin photoaging and related conditions.

This study underscores the multi-target mechanism of EHMF in preventing and treating skin photoaging, providing a strong basis for its clinical application as a dermatological agent. The experimental results not only validate the findings of our network pharmacology analysis but also highlight the intricate interplay between the various biological pathways involved in skin health. By employing rigorous experimental methodologies, we were able to demonstrate the direct effects of EHMF on cellular mechanisms associated with skin aging. These findings lend credence to our network pharmacology insights, further illustrating how EHMF modulates critical targets to mitigate the detrimental effects of UV exposure. Moreover, the experimental evidence enriches our understanding of the specific signaling pathways influenced by EHMF, reinforcing its potential as a multi-faceted therapeutic option. This integration of experimental findings with network pharmacology not only validates our hypotheses but also opens the door to further research that could explore additional applications in dermatology.

Ultimately, the convergence of experimental and computational approaches in our study highlights the importance of validating network pharmacology predictions through empirical experimentation, paving the way for more effective and targeted therapeutic strategies in the future.

## 5 Conclusion

This study demonstrates the therapeutic potential of EHMF in treating skin photoaging. UPLC-MS/MS analysis identified 22 active compounds, and network pharmacology revealed key targets including AKT1, TNF-α, STAT3, MMP9, and EGFR. Enrichment analyses highlighted the modulation of inflammatory pathways, particularly AKT and STAT3 signaling. *In vitro*, EHMF showed concentration-dependent protection in HaCaT cells, significantly reducing UV-induced damage at higher doses. *In vivo*, EHMF-H reduced wrinkles, roughness, and redness while decreasing inflammatory cytokine levels and normalizing EGFR expression. EHMF also effectively reduced phosphorylation levels of AKT and STAT3.

Overall, this study underscores the multi-target mechanism of EHMF in preventing and treating skin photoaging, providing a strong basis for its clinical application as a dermatological agent.

## Data Availability

The original contributions presented in the study are included in the article/Supplementary Material, further inquiries can be directed to the corresponding author.

## Abbreviations

HMF, Hibisci Mutabilis Folium; EHMF, extract of Hibisci Mutabilis Folium; UVA, ultraviolet A; UVB, ultraviolet B; TCM, Traditional Chinese Medicine; MTX, Methotrexate; GO, Gene Ontology; KEGG, Kyoto Encyclopedia of Genes and Genomes; UPLC-MS/MS, ultra performance liquid chromatography-tandem mass spectrometry; HaCaT, human keratinocytes; SOD, superoxide dismutase; MDA, malondialdehyde; GSH-Px, glutathione peroxidase; T-AOC, total antioxidant capacity; TNF-α, tumor necrosis factor-α; IL-6, interleukin 6; IL-17, interleukin 17; MMP, matrix metalloproteinases; DMEM, Dulbecco’s modified Eagle medium; CCK-8, cell counting kit-8.
