# Supplementary figures and images for: Comprehensive analysis of Hibisci mutabilis Folium extract’s mechanisms in alleviating UV-induced skin photoaging through enhanced network pharmacology and experimental validation
Source: Front Pharmacol. 2024 Oct 14;15:1431391. doi: 10.3389/fphar.2024.1431391 (PMC11513378; doi:10.3389/fphar.2024.1431391)

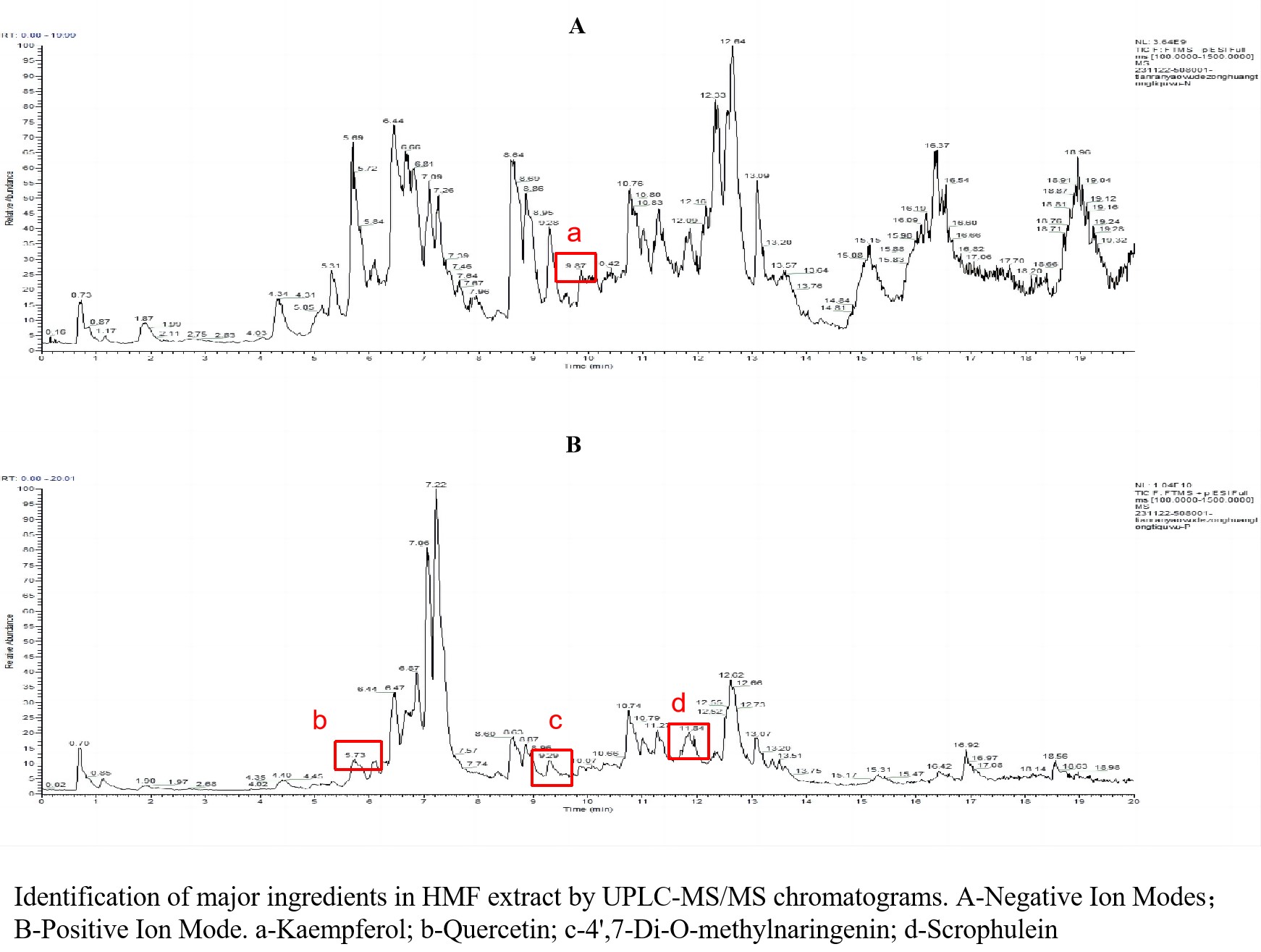

Supplement: Supplementary file 1 [file Image2.jpg]

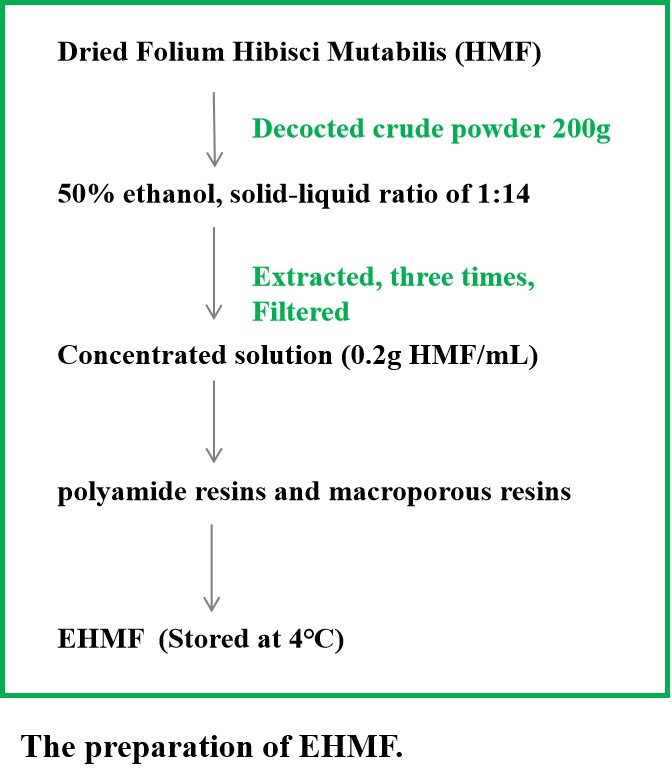

Supplement: Supplementary file 2 [file Image1.jpg]

**AKT**


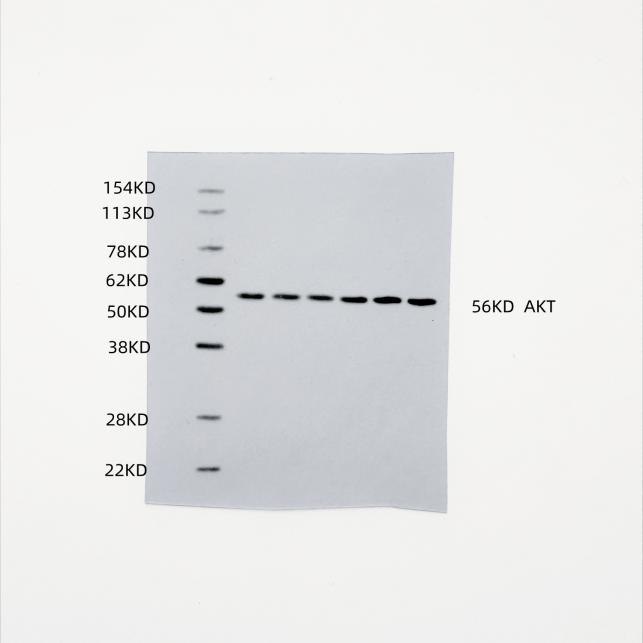

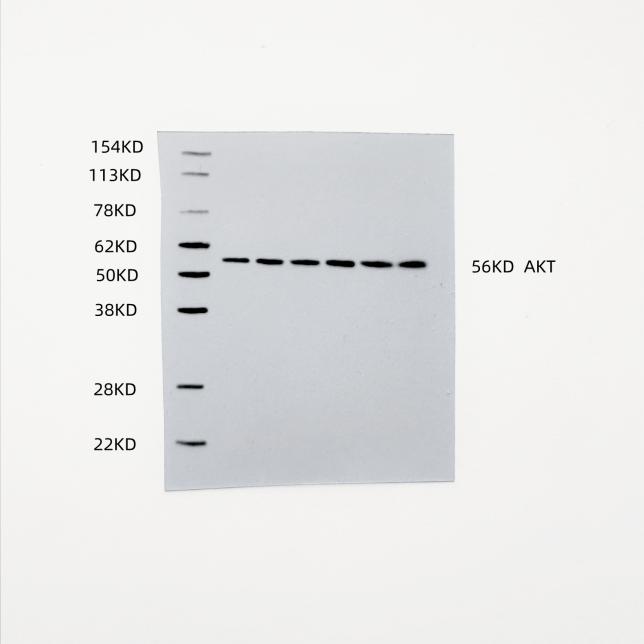


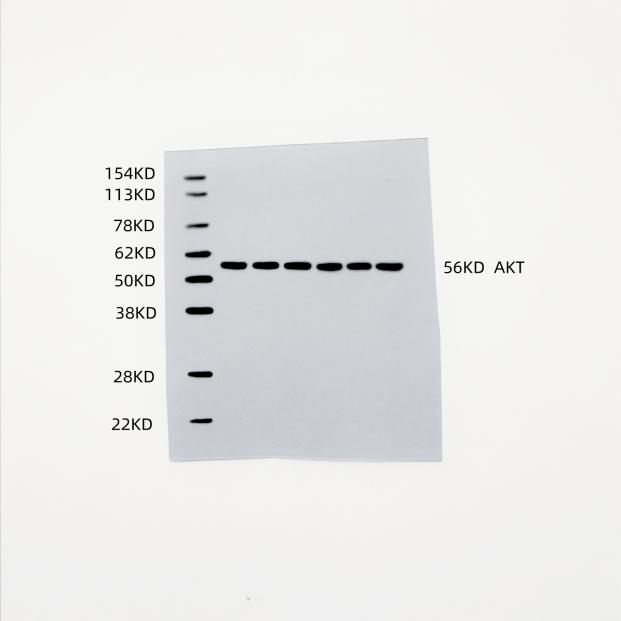


**P-AKT**


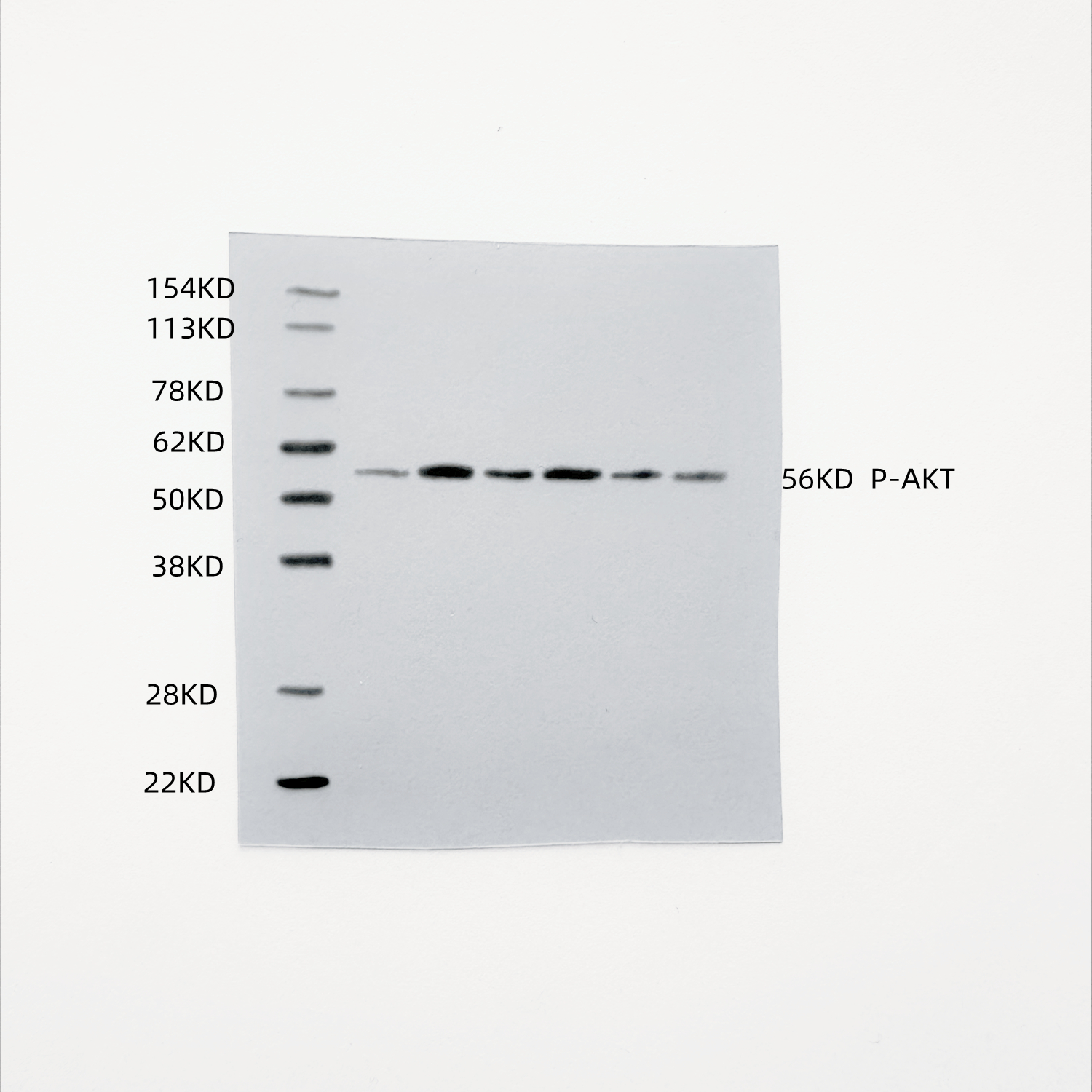


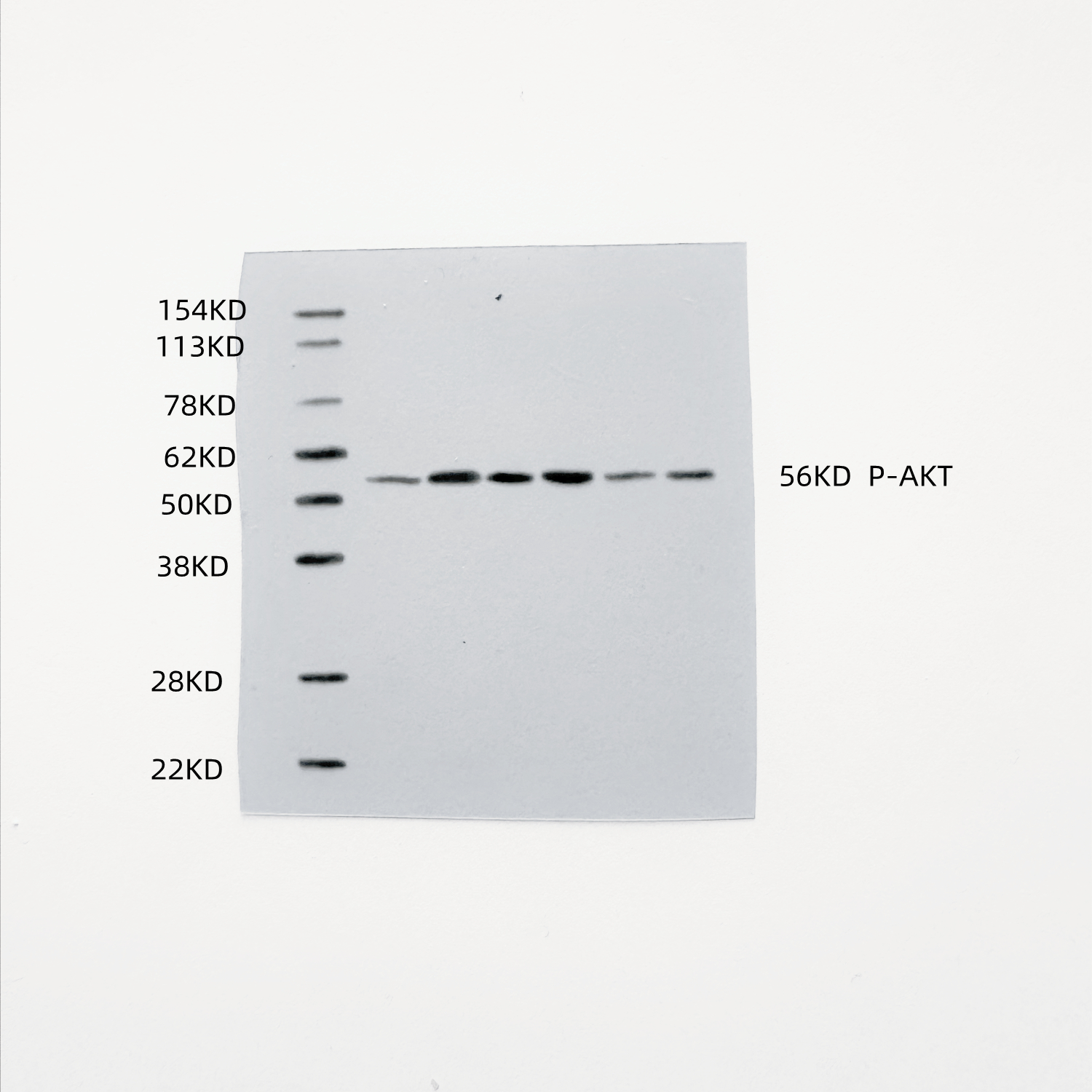


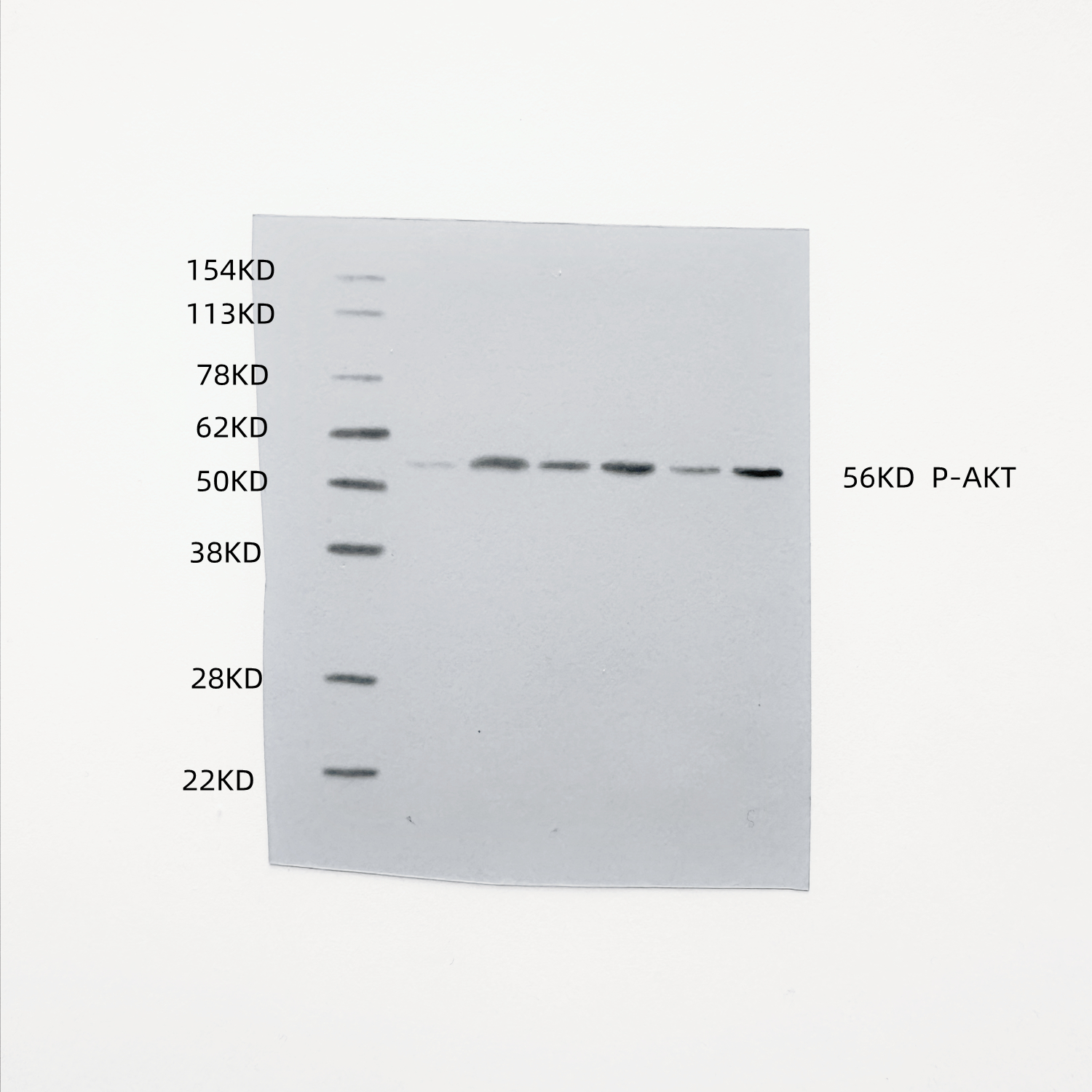


**STAT3**


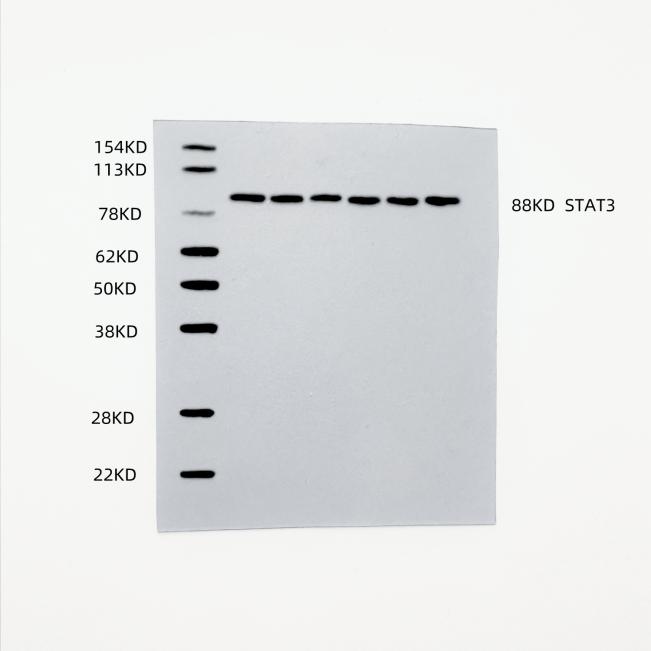

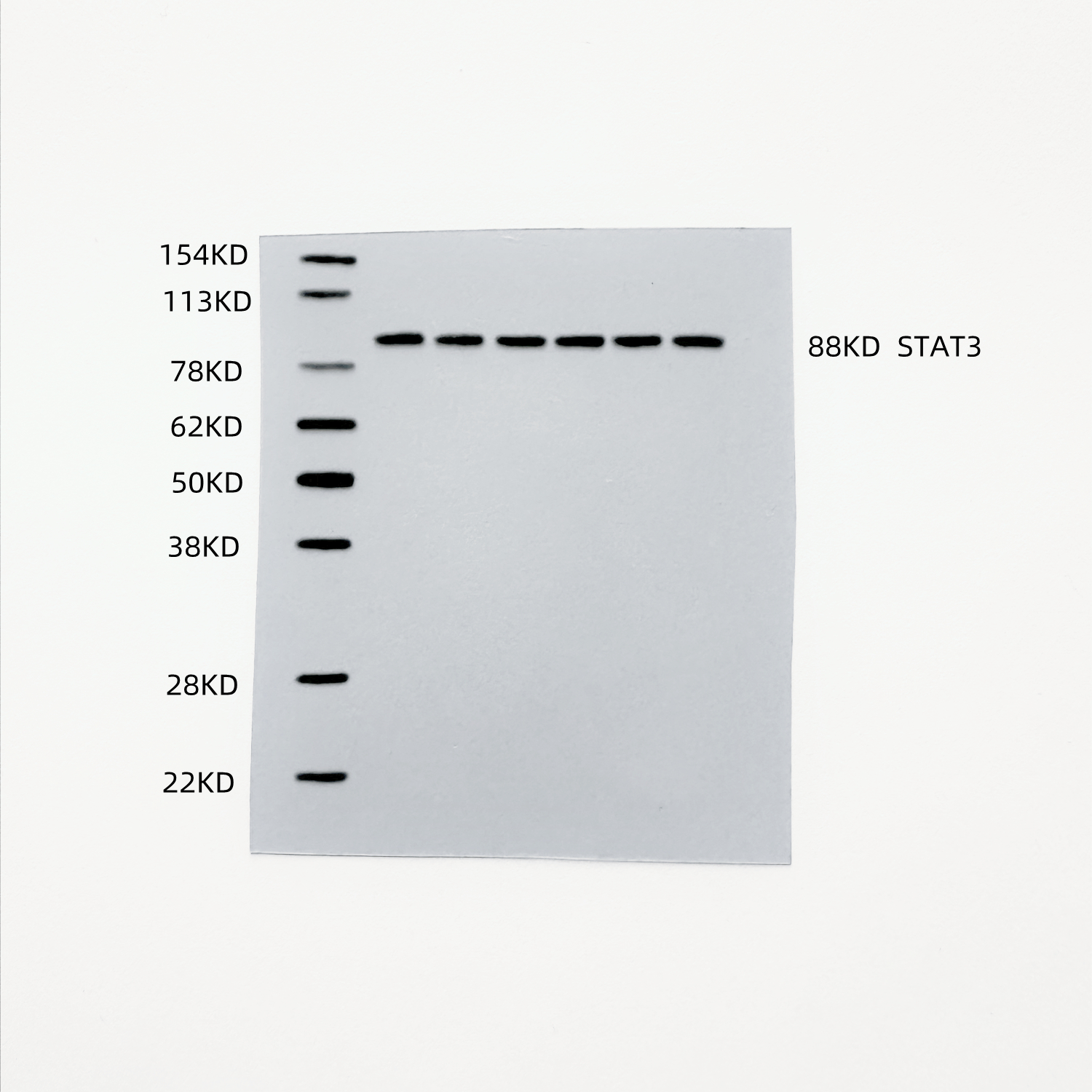


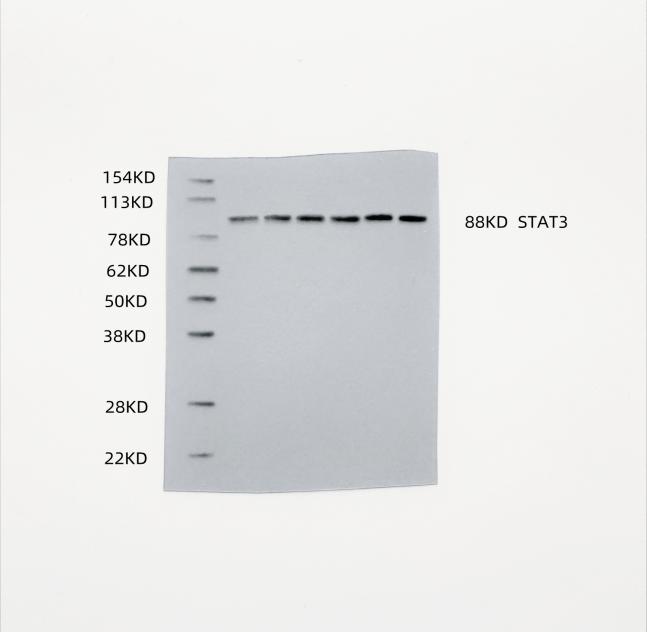


**P-STAT3**


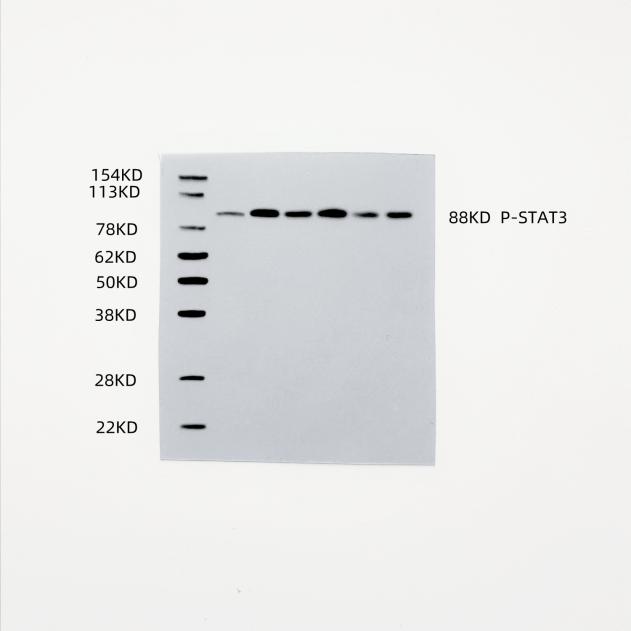


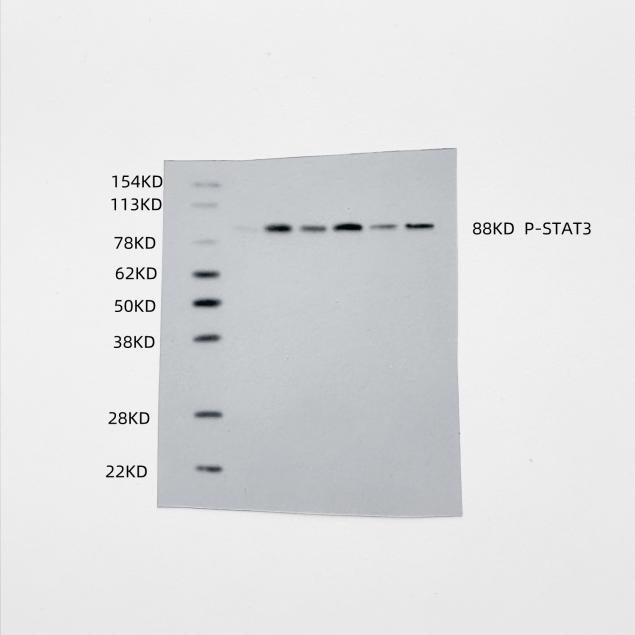


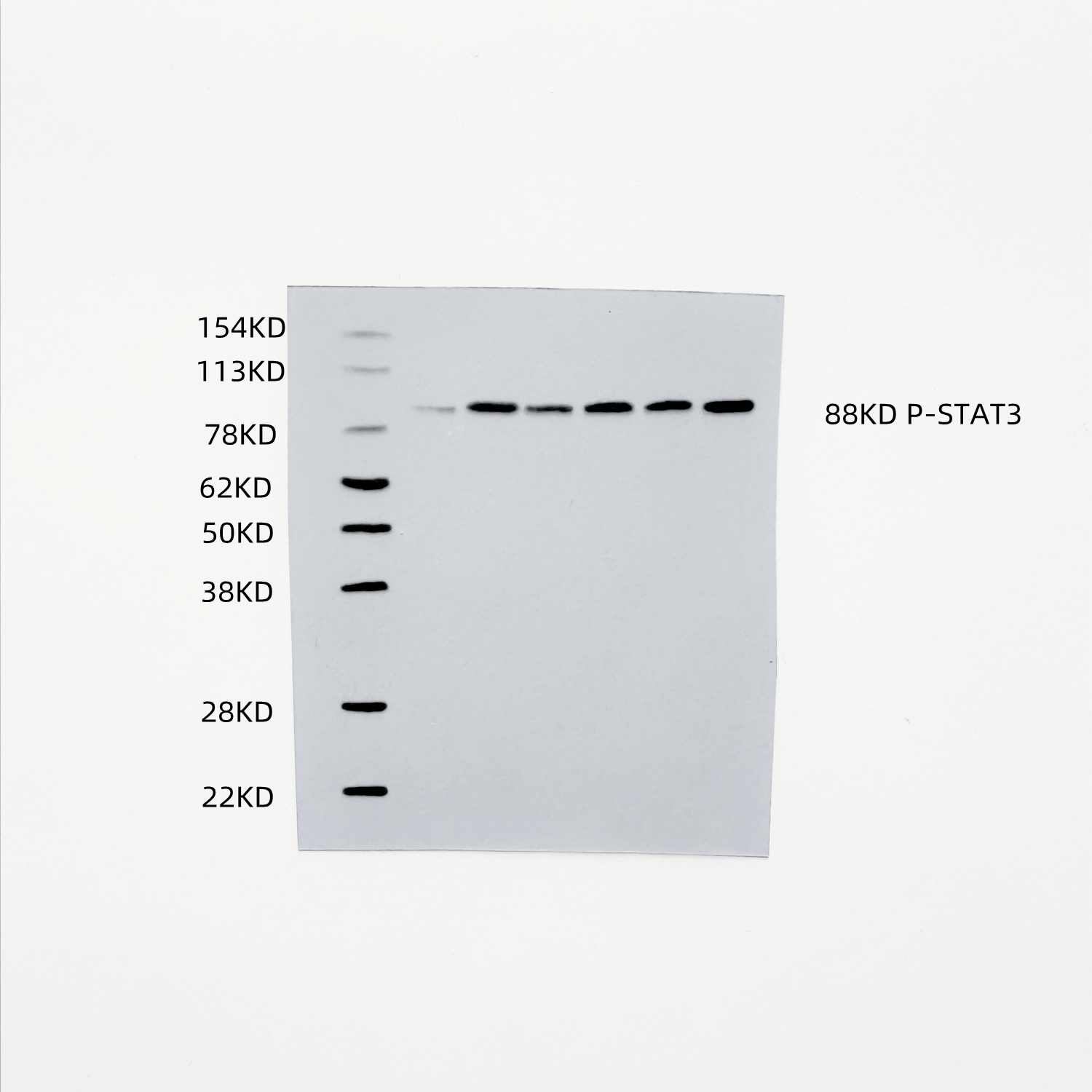


**GAPDH**


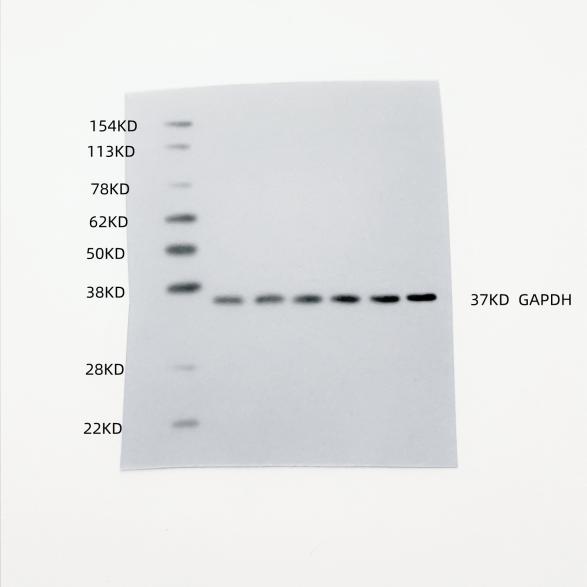


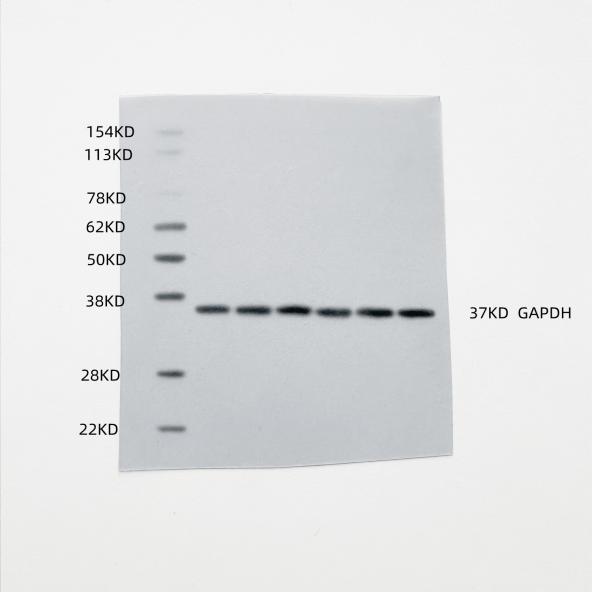


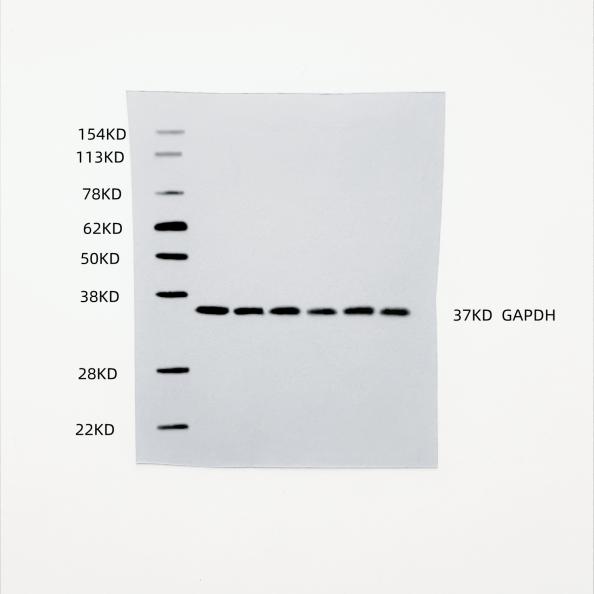

Supplement: Supplementary file 3 [file DataSheet1.docx]
